# Genetic determination of regional connectivity in modelling the spread of COVID-19 outbreak for more efficient mitigation strategies

**DOI:** 10.1038/s41598-023-34959-2

**Published:** 2023-05-25

**Authors:** Leonidas Salichos, Jonathan Warrell, Hannah Cevasco, Alvin Chung, Mark Gerstein

**Affiliations:** 1 Program in Computational Biology and Bioinformatics, Yale University, New Haven, CT 06520 USA; 2 Biological and Chemical Sciences, New York Institute of Technology, Manhattan, NY 10023 USA; 3 Department of Molecular Biophysics and Biochemistry, Yale University, New Haven, CT 06520 USA; 4 Department of Computer Science, Yale University, New Haven, CT 06520 USA; 5 Center for Biomedical Data Science, Yale University, New Haven, CT 06520 USA; 6 Department of Statistics & Data Science, Yale University, New Haven, CT 06520 USA

**Keywords:** Computational models, Genome informatics, Machine learning, Phylogeny, Infectious diseases, Viral infection

## Abstract

For the COVID-19 pandemic, viral transmission has been documented in many historical and geographical contexts. Nevertheless, few studies have explicitly modeled the spatiotemporal flow based on genetic sequences, to develop mitigation strategies. Additionally, thousands of SARS-CoV-2 genomes have been sequenced with associated records, potentially providing a rich source for such spatiotemporal analysis, an unprecedented amount during a single outbreak. Here, in a case study of seven states, we model the first wave of the outbreak by determining regional connectivity from phylogenetic sequence information (i.e. “genetic connectivity”), in addition to traditional epidemiologic and demographic parameters. Our study shows nearly all of the initial outbreak can be traced to a few lineages, rather than disconnected outbreaks, indicative of a mostly continuous initial viral flow. While the geographic distance from hotspots is initially important in the modeling, genetic connectivity becomes increasingly significant later in the first wave. Moreover, our model predicts that isolated local strategies (e.g. relying on herd immunity) can negatively impact neighboring regions, suggesting more efficient mitigation is possible with unified, cross-border interventions. Finally, our results suggest that a few targeted interventions based on connectivity can have an effect similar to that of an overall lockdown. They also suggest that while successful lockdowns are very effective in mitigating an outbreak, less disciplined lockdowns quickly decrease in effectiveness. Our study provides a framework for combining phylodynamic and computational methods to identify targeted interventions.

## Introduction

As of October 2021, two years since the start of the pandemic, coronavirus disease 2019 (COVID-19)-related deaths have surpassed 4,800,000 worldwide and 700,000 in the United States. Due to the severity of the pandemic combined with the advent of sequencing technologies, the amount of sequencing data within such a short time period for a single outbreak is unprecedented. Indeed, many resources are available for COVID-19 genome research, including GenBank, GISAID, and Nextstrain^1–3^. GISAID is currently the largest COVID-19 database with more than 3,900,000 severe acute respiratory syndrome coronavirus 2 (SARS-CoV-2) complete genomes^2^. This number surpasses the number of human immunodeficiency virus or hepatitis C virus sequences in the Los Alamos national database^4,5^ and far exceeds the 1,760 sequences of influenza A/H3N2 collected from 2013 to 2020. The COVID-19 genomes in the GISAID database represent the spread of the pandemic from China to 188 countries worldwide, with more sequences added every day.

Recent studies have primarily modelled the transmission, diversity and spatial phylogeography of SARS-CoV-2 in a historical context^6–12^. According to these studies, with respect to the United States, COVID-19 first arrived in Washington^11^, in what was considered a cryptic infection^11,13^. However, cases in individuals with no relevant travel history also occurred in California in late January/early February^11^.While the first lineages in Washington and California originated from China, subsequent infectious lineages [notably in New York (NY)] appeared to derive from Europe^11,14^. In this context, early results also suggested that multiple worldwide transmissions were responsible for the outbreak in the North-East of United States^12^.

From the beginning of the pandemic, researchers have developed various approaches for the modeling of the outbreak, using either epidemiological or demographic data^15–20^. Numerous -sometimes contradicting- prediction models have offered temporal, and locally isolated results based on local outbreaks^16–18,21,22^, while individual countries have implemented their strategy to combat the outbreak, including controversial approaches such as “herd immunity”^23–25^. At the same time, local regions have applied different forms of lockdowns in an attempt to mitigate viral spread with non-pharmaceutical interventions (NPI)^22,26–28^. While most previous studies have offered valuable insights into the history of viral transmissions and the effectiveness of locally implemented NPIs, they tend to overlook the inland spread of the virus, which we term ‘aggregated transmission’ as a measure of viral flow, to provide a unified mitigation strategy that complements local implementations. Notably, shortly before the pandemic, Dellicour et al. demonstrated the impact of barriers on dispersal frequency for the West African Ebola virus outbreak, while quantifying the spread’s velocity and hypothetical impact under a distance-dependent diffusion model^29^.

In this study, we use the initial SARS-CoV-2 wave in United States as a testbed for our models. During our test period, the spread by air travel was limited, while the virus spreads to both previously infected and uninfected regions. First, we show that most of the outbreak derived from few phylogenetic lineages rather than random disconnected outbreaks, depicting a mostly continuous viral flow. We also demonstrate a strong association between the temporal and geographical spread of the virus. By using a case study of seven states [New York (NY), New Jersey (NJ), Connecticut (CT), Massachusetts (MA), Pennsylvania (PA), Maryland (MD) and Virginia (VA)], we introduce the concepts of ingrowing, incoming and outgoing genetic connectivity between states and regions as factors that influence the geographic spread. We then apply regression and random walk models to illustrate the importance of these factors combined with epidemiological and demographic factors -such as virus reproduction rates and the Urbanization Index to provide more informative predictions and to explain the temporal and geographic spread of the pandemic. By modeling the viral aggregated transmission through geographical routes and regional connectivity, we reveal broader implications and opportunities to consider more efficient mitigation strategies for slowing viral migration with unified, cross-border selective interventions.

### Major outbreaks linked with few European lineages showing viral flow continuity

**Figure 1:**
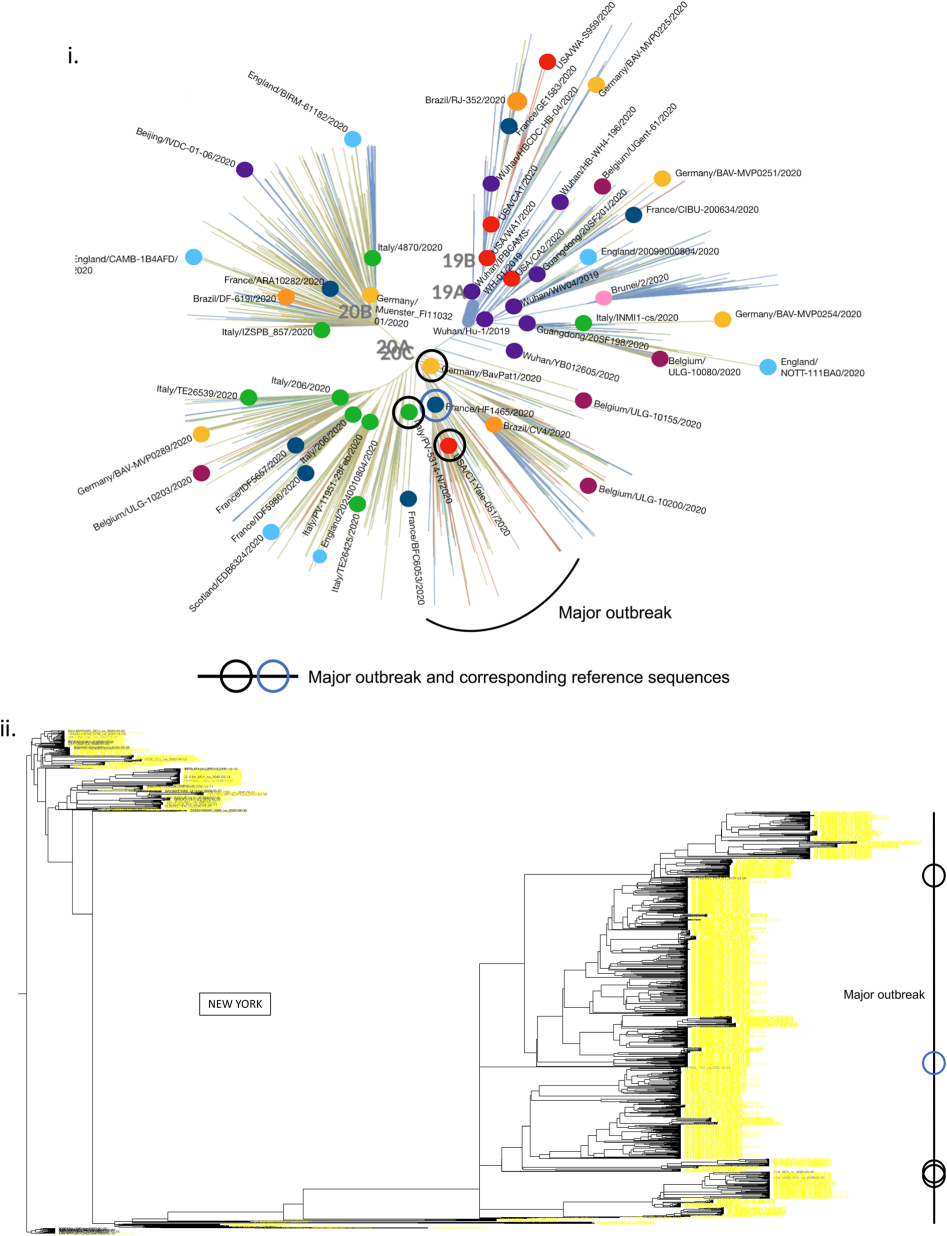

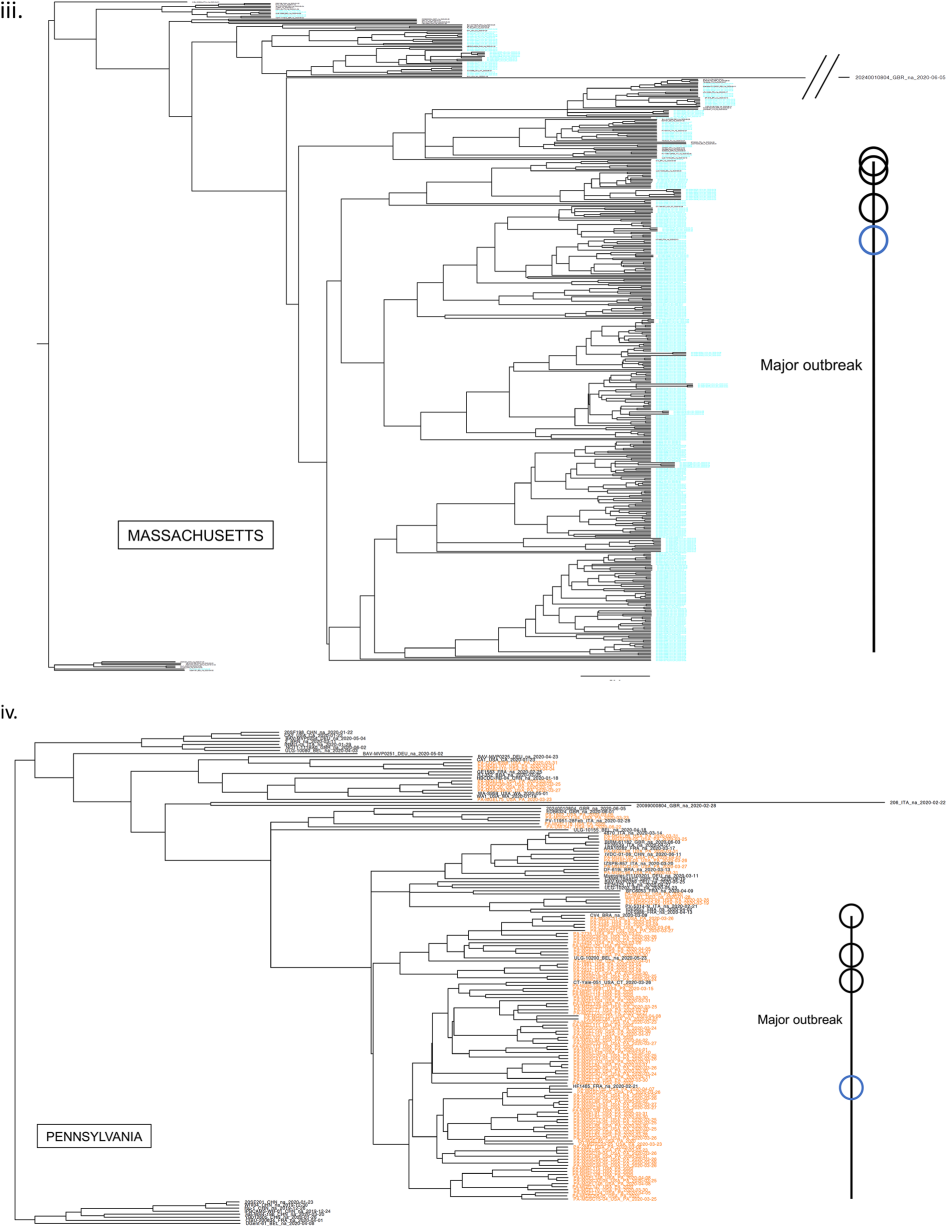

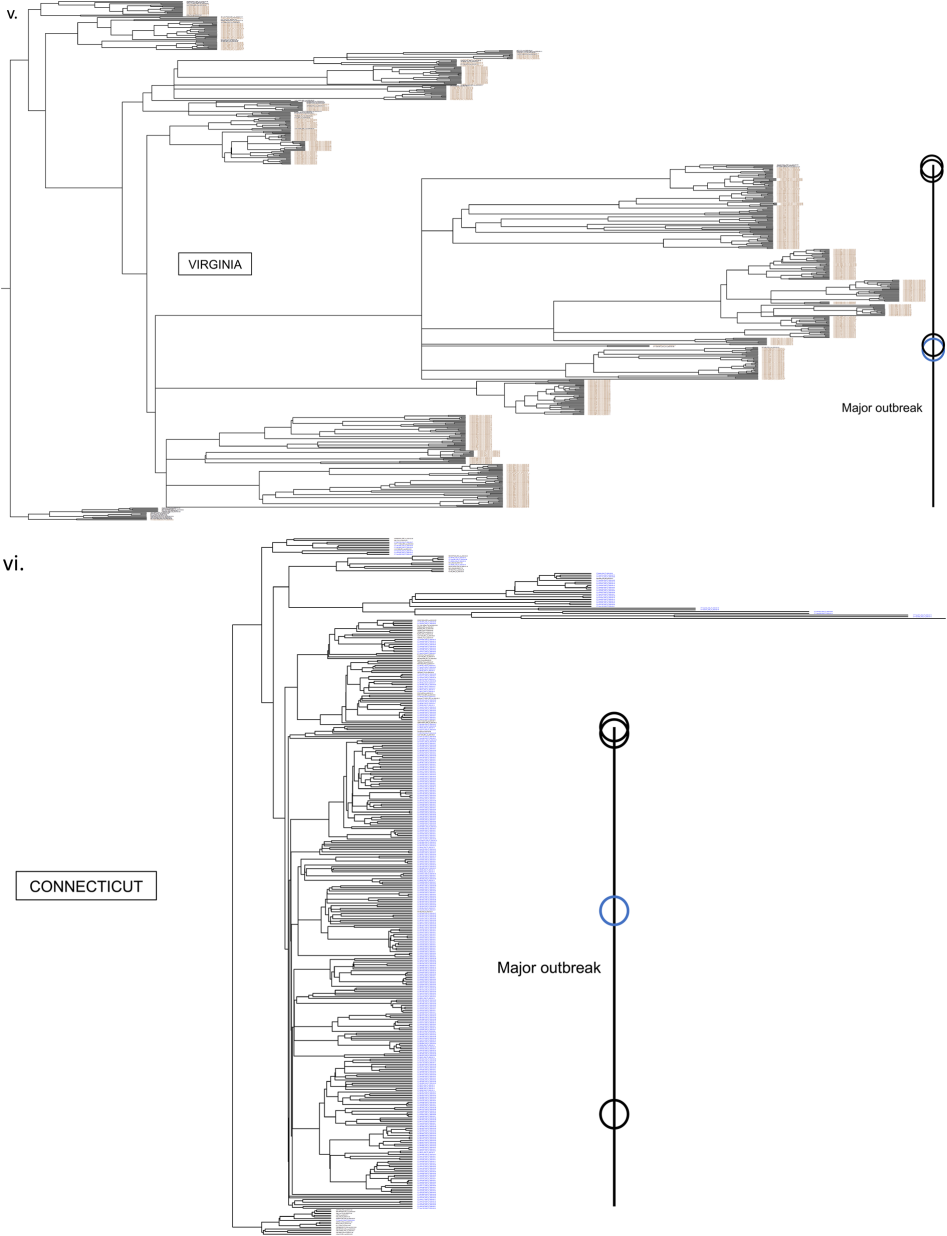

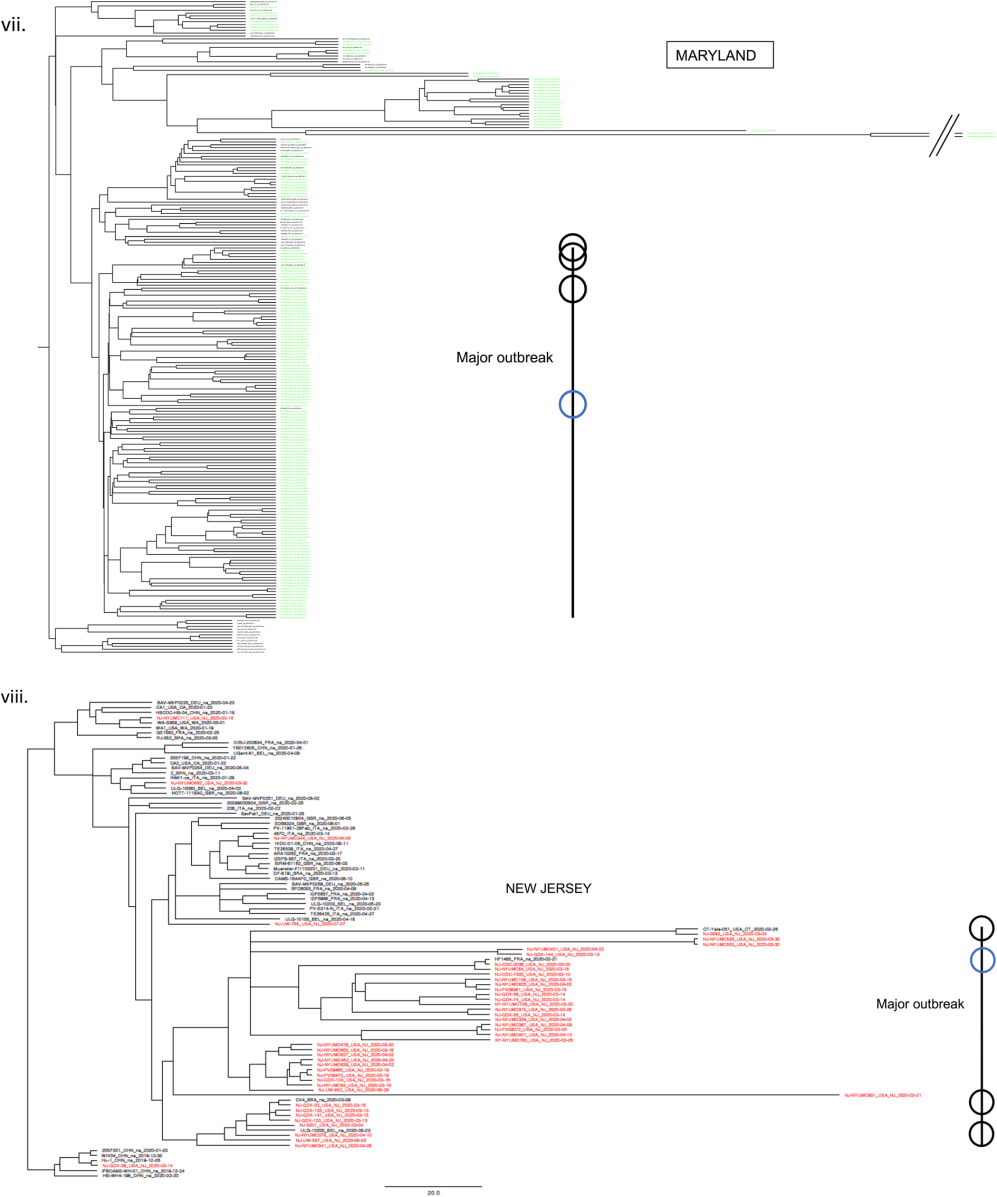
Few worldwide infections responsible for major and minor outbreaks in 7 states. According to a Nextstrain tree, initially there were five main initial lineages of the pandemic (19A, 19B, 20A, 20B, 20C), which can be used to suggest the original routes of the transmission in the United States. In (**i**) we show the topology of our selected world reference sequences as collected spanning the Nextstrain tree (https://github.com/nextstrain/ncov) on June 25th (dots on tree topology). From these randomly collected sequences, sample HF1465 FRA (blue circle) is the sequence that consistently clusters with each state’s major outbreak (black line). Three other reference sequences from Italy, USA, and Germany (black circles) cluster -again consistently- with each state’s major outbreak (black line), suggesting that most of the outbreak derives from these lineages, which correspond to a small part of the whole Nextstrain tree topology. In (**ii**) we show the New York outbreak, which we consider to be the outbreak epicenter. In (**iii–viii**) we show the phylogenetic tree analysis for Massachusetts, Pennsylvania, Virginia, Connecticut, Maryland, and New Jersey, as rooted by the older lineage that contains sequences from Wuhan dating in 2019. Black lines represent each states major outbreak, while the four circles on the black line correspond to the 4 specific reference sequences from a single initial lineage. Tree in Fig. (**i**) was inferred using *nextstrain/ncov *(https://github.com/nextstrain/ncov). Trees in figures (**ii–viii**) were visualized using FigTree v1.4.4. Figure 1 was designed and illustrated with PowerPoint 2019.

While currently consisted of millions of sequences, by the end of April 2020, the GISAID database contained only about 3500 complete SARS-CoV-2 genomes. For modeling the first wave of the COVID-19 outbreak in our case study of 7 states, we also collected all 1,505, 353, 418, 45, 112, 178, and 522 sequences from NY, CT, MA, NJ, PA, MD, and VA, respectively, that were collected between January 29, 2020 and July 05, 2020, for a total of 3133 sequences. To create a dataset of world reference sequences, we sampled 50 early SARS-CoV-2 sequences mostly representing the backbone of all five COVID-19 lineages as determined initially by the state-of-the-art Nextstrain tree^3^, while also including some later tips for calibration^30^. Lineages 19A and 19B represent the earliest detected infections, which were closely associated with the Wuhan epidemic. These clades were also subsequently used to root the phylogenetic trees (for presentation purposes), in the absence of an appropriate outgroup^31^. Then, we inferred a Bayesian phylogenetic tree using BEAST, which includes the consideration of population growth models, as commonly implemented in viral phylodynamic analyses. For all states, the major outbreak clustered with a specific European lineage (represented by reference sequence: HF1465 FRA). For NY, CT, and MA this lineage clearly constituted the dominant outbreak. For NJ, PA, MD and VA, a significant secondary outbreak -which also circulates in NY, CT, and MA- occurred (Fig. 1, Figs. s1–s7). Our results demonstrate the outbreak’s continuity and regional connectivity despite multiple initial worldwide transmissions.

### Assessing genetic connectivity between states from phylogenetic information

**Figure 2:**
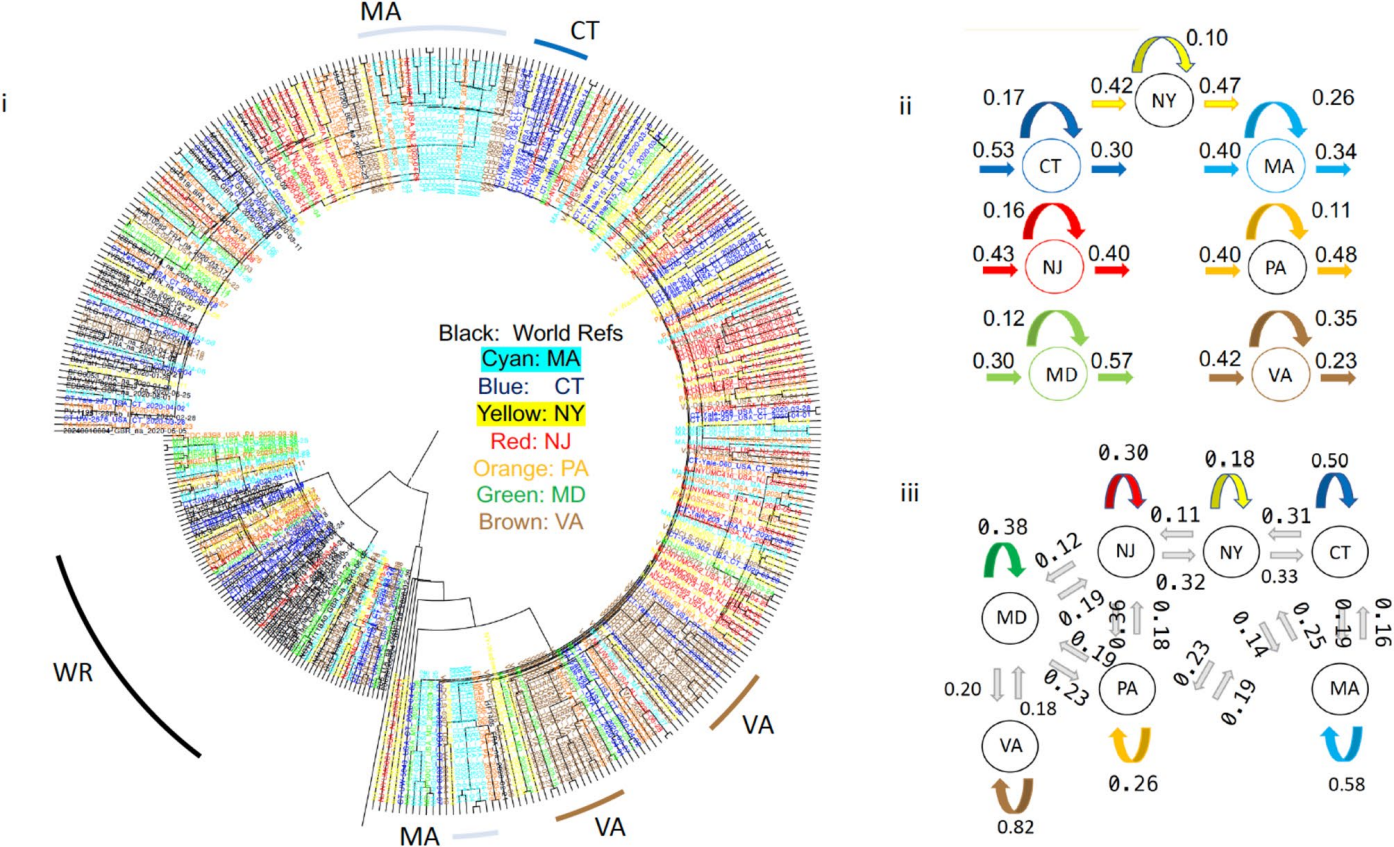
Assessing Genetic Connectivity Between States from phylogenetic information. In (**i**) using world reference sequences and selected reference sequences from 7 states, we inferred a phylogenetic tree with time constraints for each state. Each sequence’s tip color corresponds to the state it was collected from. Using pairing and dating information, we derived (**ii**) incoming, outgoing, and ingrowing connectivity for each state and (**iii**) genetic connectivity between all states. For convenience, we only show neighboring and geographical connectivity. Tree in figure (**i**) was visualized using FigTree v1.4.4. Figure 2 was designed and illustrated using PowerPoint 2019.

By selecting a subset of (whenever possible) 50 reference sequences per state (in addition to the set of world reference sequences), we built a phylogenetic tree that includes sequences from all seven states. We then inferred a connectivity map between the states by parsing the tree’s partitions with respect to each state’s prevalence (see “Methods”). The connectivity map does not represent direct viral transmissions between individuals, but rather the genetic connectivity between states X and Y as a rate or probability that sequences from these regions are grouped together in the tree’s considered partitions, while using dating information to further rank the sequences and assign directionality between terminal pairs. This allows us to assess incoming, outgoing, and ingrowing rates for each state (Fig. 2ii), as well as directional connectivity between states (Fig. 2iii). Overall, the NY outbreak showed the highest connectivity compared to other states, while VA and MA outbreaks showed the lowest connectivity. Interestingly, although CT showed a high connectivity comparable to that of NJ, the decreased number of outgoing versus incoming connections explains the low connectivity exhibited by MA. This is also supported by the outbreak’s high connectivity from NY to CT (NY → CT) rendering CT as a potential bottleneck (Fig. 2).

### A baseline model for modeling viral flow using factors associated with early spread

**Figure 3:**
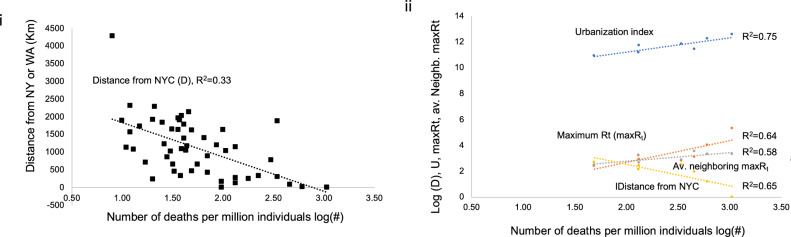
Distance from hotspots correlates with initial outbreak severity. Using data collected on the 29th of April we show the logarithmic association between i) the number of deaths per million individuals for every state and the distance from hotspots (New York or Washington). In a case study of 7 states (New York, New Jersey, Connecticut, Massachusetts, Pennsylvania, Virginia, and Maryland), ii) we show the logarithmic association between the number of deaths per million individuals versus the Distance from New York city, each state’s maximum reproduction rate Rt, each state’s average neighboring maximum Rt, and each state’s urbanization index.

**Figure 4:**
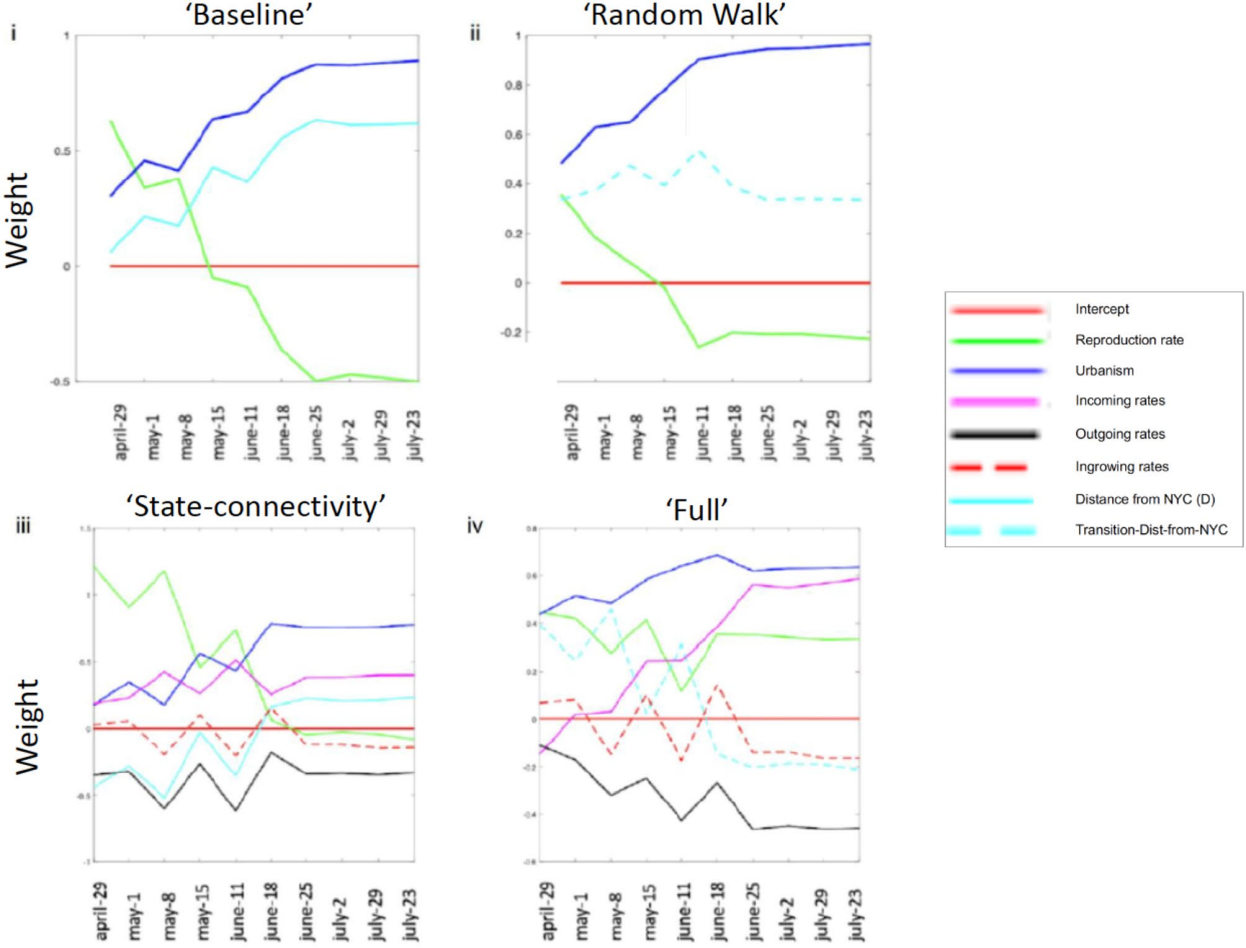
Four predictive models with increasing complexity to examine the role of genetic connectivity. Predictive models with connectivity-based features. (i,ii) baseline and “random walk” models respectively (three factors), (iii,iv) “state connectivity” and “full” models respectively (six factors). Likelihood significance was found for models “random walk” vs “baseline”, and “full” versus “state connectivity. (p = 0.0003, 0.0273 resp., 2-sided t-test for Pearson’s r). Model fit ${R}^{2}$ values are: 0.850 (Baseline), 0.877 (Random walk), 0.956 (State-connectivity), 0.967 (Full).

After assessing the genetic connectivity between states, we want to evaluate its role in the spread and severity of COVID-19, compared to a simple outbreak prediction model (the baseline model). Initially, we aimed to identify factors associated with the early geographic spread and severity of the outbreak. On average, deaths occurring on the 29th of April typically follow 5.1 days of incubation and 13 days from symptoms^32^. Using the numbers of ‘deaths per 1 million population’ as a proxy for regional outbreak severity, we first assessed the association between distance from initial viral hotspots and the severity of the viral outbreak across United States. Introducing New York City (NYC) as a single initial hotspot, showed a high negative correlation (*r* = − 0.37, p-value = 0.008) between the severity of the outbreak and the distance from hotspot. When we included Seattle (or San Francisco) as a second hotspot, the association strengthened (*r* = − 0.43, p-value = 0.001). Finally, when fitting a logarithmic curve, the association increased further to R^2^ = 0.33 (Fig. 3). These results suggest a strong association between the outbreak’s severity during the first wave and the distance from the two initial hotspots. By limiting our study to the case study of seven states (NY, CT, MA, NJ, PA, MD, and VA), we established an even stronger relationship between the distance from NYC and the severity of the initial spread. Additional factors were also strongly associated with the spread and severity of the outbreak at the beginning of the first wave, including urbanism, maximum effective reproduction rate Rt per state, and average maximum Rt from neighboring states. Thus, for our simplest baseline model (Fig. 4i) we included urbanism (Urbanization index U), geographic distance from the hotspot (D), and maximum virus reproduction rate Rt (maxRt). In a network flow context, these factors can be considered analogues to node capacity, connectivity, and network sources respectively^33^. Moreover, we found that these factors were strongly associated with the outbreak’s severity, explaining more than 75% of the variance (Fig. 3ii).

### Three additional models show increased predictive accuracy by including genetic connectivity

We next tested the importance of various features in predicting the per-state death rate across the first wave of the pandemic (March to August 2020) with respect to aggregated transmissions. To determine the importance of regional genetic connectivity in explaining and predicting the outbreak intensity throughout the entire first wave, we built three additional regression models (beyond our baseline model) with increasing complexity, combining phylogenetic information with epidemiological data from ten dates (April 29, May 1, 8, and 15, June 11, 18 25, July 2, 29, August 23). Our analysis included the estimated incoming, outgoing, and ingrowing rates for each state (see Fig. 2ii), and the transmission-based distance from NY as constructed from the directional connectivity rates (see Fig. 2iii). The full feature set comprised the maximum reproduction rate per state (R_t_), urbanization index (U), geographic or genetic-based transmissional-distance from NY (D or D_t_ respectively), and incoming, outgoing, and ingrowing transmission rates.

Beyond our baseline, in our second model (“random walk”, Fig. 4ii), we substituted D with the transmissional-distance (D_t_), a proxy for viral flow, using a random walk between the states, based on genetic connectivity (see “Methods”, Fig. 2iii). By including transmissional-distance (D_t_), we were able to significantly increase our model’s predictive power throughout the first wave compared to our baseline model (p = 0.0003, Fig. s8). In our third model (“state connectivity”, Fig. 4iii), we returned to using the geographic distance D, but in this case, we also included the total incoming, outgoing, and ingrowing rates for each state, also estimated using genetic connectivity (see Fig. 2ii). Finally, in our fourth model (“full”, Fig. 4iv), we again replaced D with D_t_, while still including the states’ incoming, and outgoing rates. While our fourth model integrates genetic connectivity in D_t_, this information derives from the tree partitions and is also used in calculating the incoming, outgoing, and ingrowing connectivity for each state. Therefore, D_t_, the incoming, and outgoing rates often behave in a complementary manner. However, our “full” model is still significantly more informative than the “state connectivity” model, which does not include the transmissional-distance (p = 0.0273). Moreover, our full model indicates that the initial importance of D or maxR_t_ during the beginning of the outbreak, is gradually replaced by the state’s connectivity rate, as the outbreak spreads away from the initial hotspots. The model fit ${R}^{2}$ values for our models are: 0.850 (Baseline), 0.877 (Random walk), 0.956 (State-connectivity) and 0.967 (Full). While, these values are extremely high, it should be mentioned that this is a case study which is limited to only seven states. Overall, U and D showed high significance throughout the entire first wave, while the use of maxR_t_ showed greater significance at the beginning of the outbreak but eventually decreased (Fig. 4, Fig. s8). This is possibly because maxR_t_ represents the virus reproductive rate for only the first stage of the outbreak (i.e. in March–April), before the implementation of lockdowns.

### Managing regional connectivity for targeted mitigation strategies

**Figure 5:**
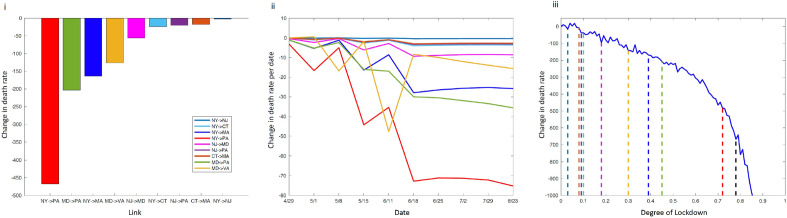
The total and temporal effect in saving lives by restricting state pair connections. We estimated the sum of total lives that would be saved if we restrict viral connectivity by 90% between geographic link between two states. In (i) we show the total number of lives saved per million individuals per case, while in (ii) we show the temporal distribution of these deaths, showing when specific links become important. In (iii) we show the association between the change in death rate and various degrees of full lockdown (in terms of efficiency). Colored dotted lines correspond to a 90% blocking degree for each link, indicating the degree of full lockdown with an equivalent effect. The black dotted line corresponds to targeting the two most important links.

After assessing the significant role of genetic connectivity on predicting the spread and severity of the outbreak, we tested the positive or negative impact of selective mitigation interventions by removing the connections between state pairs. These interventions may represent different implementations such as blockades between states, enhanced vaccination etc. Our “random walk” model which considers transmissional-distance D_t_ calculated from these paired connectivities, was significantly more predictive than our baseline model (p = 0.0003). Using our implementation of the “random walk” model, we re-predicted the total number of deaths by modeling severely restricted mobility—in particular, by systematically reducing the connectivity between every geographically linked state pair according to Fig. 2iii by 90%. Our results suggested that by restricting mobility by 90% between NY from PA, as well as MD from PA would save approximately 450 and 200 lives per million individuals respectively, after the lockdowns (Fig. 5i). This finding is particularly interesting since our model appears to consider the drop in deaths in specific states after the imposed lockdowns (based on epidemiological data from NJ, NY, MA, and CT) and respond to the temporal flow of the pandemic which resulted in later death peaks in states like VA. This trend becomes more evident in Fig. 5ii, where we depict the temporal effect of each blockade in reducing the number of total deaths per million individuals at each time point. The link between NY and PA becomes important around May 2020, while the link between MD and PA is important a month later. At the same time, by estimating the change in death rate for various degrees of full lockdown (in terms of efficiency), we found that by targeting the two most important links (NY > PA and MD > PA) we were able to achieve the effect of a full lockdown with 78% efficiency. Finally, our results show that while strict full lockdowns (> 90% efficiency) perform well in our models (in terms of change in death rates), the effects of less efficient lockdowns (< 80%) can be potentially be achieved by targeted interventions (Fig. 5iii).

## Discussion

Previous studies have provided an important historical view of the travel and viral transmission of COVID-19 based on genetic variability^6,8,9,11–15^ Click or tap here to enter text. Other data-driven work has modeled the spread of SARS-CoV-2 and the effectiveness of government interventions^20,27,28^. Thus far, the most efficient NPIs are forms of regional lockdowns^19,22,26,35^, while strategies relying on ‘herd immunity’ have been disputed^23–25^.

Here, we used SARS-CoV-2 genomes to determine regional connectivity as a direct measurement of the viral mobility in a case study of seven states. We generated and applied four regression models to evaluate the importance of different factors influencing outbreak severity throughout the first viral wave. Our models showed high predictability and temporal variation.

Our results can explain the discordance among regions and strategies, especially between the first and second pandemic waves. For example, states at a larger distance from hotspots are able to handle a milder initial outbreak before the virus becomes established at a later time point, depending on the transmissional-distance (i.e. the speed of the wave) and regional connectivity. Similarly, states with lower connectivity (e.g. naturally or physically isolated regions) are more efficient in battling the viral spread, as they encounter a reduced viral wave and fewer incoming infections. This finding suggests that reducing incoming transmission routes can have a significant effect. This approach does not necessarily require complete isolation, but rather a restriction of specific viral transmission routes. Moreover, our framework can also be used to implement enhanced vaccination strategies, for instance, by targeting specific transmission routes we can drastically reduce the temporal spread of an outbreak. Finally, our results also suggest that states deciding to follow less stringent mitigation strategies are largely responsible for their outgoing viral connectivity, affecting neighboring regions, while in turn taking advantage of the low incoming connectivity resulting from potential neighboring lockdowns.

Throughout our analyses, we aimed to model the viral geographic aggregated transmission as a probability rate, rather than a compilation of individual transmissions. To showcase our models, we restricted our analyses during the first months of the pandemic where commercial air-travel was limited. Therefore, we focused on estimating the genetic connectivity between adjacent states and inland viral spread, although we may therefore have missed non-adjacent state infections (e.g. private planes, trains etc.). Nonetheless, our connectivity network can be expanded to consider genetic connectivities between non-adjacent states. Similarly, for reasons of convenience and simplicity, we trained our models using a subset of around fifty SARS-CoV-2 sequences per state. Again, our framework is set to include varying number of sequences per region, according to their respective prevalence and regional sampling (see “Methods”). Finally, according to our analysis, the first wave of the outbreak derived from few viral lineages, which allowed us to -in principle- model a homogenous viral outbreak. In the presence of parallel outbreaks, our framework can also be implemented with respect to those outbreaks and/or the geographic aggregated transmission of specific strains. While we were able to identify and quantify factors that contribute to the viral spread within the specific framework of initial COVID-19 outbreak, the implementation of predictive models in the future would require further parametrization and validation using new viral sequences or simulations (we note that constructing such simulations for validation is a challenging task, since the effects of unmodelled variables may bias the results; indeed, a particular strength of our framework is that the genetic connectivity measure implicitly incorporates the influence of such latent variables).

By deriving genetic connectivity among regions from genomic information, we create a model and a proxy for the flow of the viral wave using factors that have temporally influenced the severity of local outbreaks throughout the pandemic. We then applied this model to consider the outcome of selective intervention strategies using geographic blockades. Overall, our results suggest that unified mitigation strategies are more efficient for responding to a pandemic. This study also provides a framework for pursuing these strategies, which can be implemented for both pharmaceutical interventions (e.g. vaccinations) and NPIs (e.g. lockdowns, blockades).

## Methods

### Available sequences

The datasets used in this study are available in public databases. SARS-CoV-2 genomes were retrieved from the GISAID database^2^. For the first wave of the COVID-19 outbreak in the United States, we collected 1505, 353, 418, 45, 112, 178 and 522 sequences from NY, CT, MA, NJ, PA, MD, and VA, respectively, that were sampled between January 29, 2020 and July 05, 2020, for a total of 3,133 sequences.

#### ^237^World reference sequences

To apply reference sequences representing the global pandemic, we manually selected 50 sequences spanning all Nextstrain lineages 19A, 19B, 20A, 20B, and 20C (see Fig. 2i). The majority (76%) of the selected world reference sequences represent early infections, occurring between December 2019 and April 2020 in order to consider the pandemic’s early divergence profile and correspond to the backbone of a tree analysis. The rest of the sequences were selected to be used as tip calibration^30^. More specifically, our world reference sequences include 3 sequences from December 2019, 8 sequences from January, 6 sequences from February, 10 sequences from March, 10 sequences from April, 6 sequences from May, and 6 sequences from June 2020 (Supplementary Fig. s9). By including the world reference sequences, our goal is not to subtype the individual transmissions in different states, but to determine whether neighboring states had similar outbreak profiles when inferring their individual state trees.

#### State reference sequences

We randomly selected up to 50 reference sequences from each state, prioritizing the selection of one sequence per bipartition with higher than 50% posterior probability. Excluding world reference sequences, we selected 50 sequences from NY, CT, MA, and VA, and 43, 37, 22 from NJ, PA, and MD, respectively. To demonstrate that there is no potential accidental bias, we show the position of these sequences spanning the state trees of NY, CT, MA and VA (Supplementary Figs. s10–s13) and we also calculated the number of base differences per sequence from averaging over all sequence pairs. Standard error estimates were obtained by a bootstrap procedure of 100 replicates (Supplementary Fig. s14). Evolutionary analyses were conducted in MEGA11^38^.

### Phylodynamic analysis

By retrieving the genomic sequences from GISAID (Data s1–7), we used the “-auto” with less than 2000 sequences command for MAFFT.v7^39^ to produce multiple sequence alignments for every state based on nucleotide sequence data. Then, using BEAST v.2.6.339^41^, we performed Bayesian phylogenetic analysis with time constraints, under a generalized time reversible evolutionary model with invariant sites. To determine the appropriate growth models and population size, we tested various growth models including (i) Yule process, (ii) exponential growth, (iii) logistic growth, (iv) Bayesian skyline, and (v) birth–death skyline models with chain lengths of 100 million states while using 20% as our burn-in and sampling of 10,000 trees. We evaluated the efficacy of these models using Tracer v1.7.1(46). The best model (Yule process) for this data was selected based on the effective sample size (ESS > 200) on tree posterior and prior, trace inspection, and demographic data. None of the remaining growth models produced a converging trace for the mixed tree with combined states (Fig. 2). For our best model, MCMC was allowed to run for a chain length of 300 million states, while using 20% as our burn-in states and sampling aiming for 10,000 trees. The best tree was inferred using TreeAnnotator v2.6.3 from BEAST suite, while selecting for maximum clade credibility and “Common ancestor heights”.

### Estimating transmissional genetic connectivity

**Figure 6:**
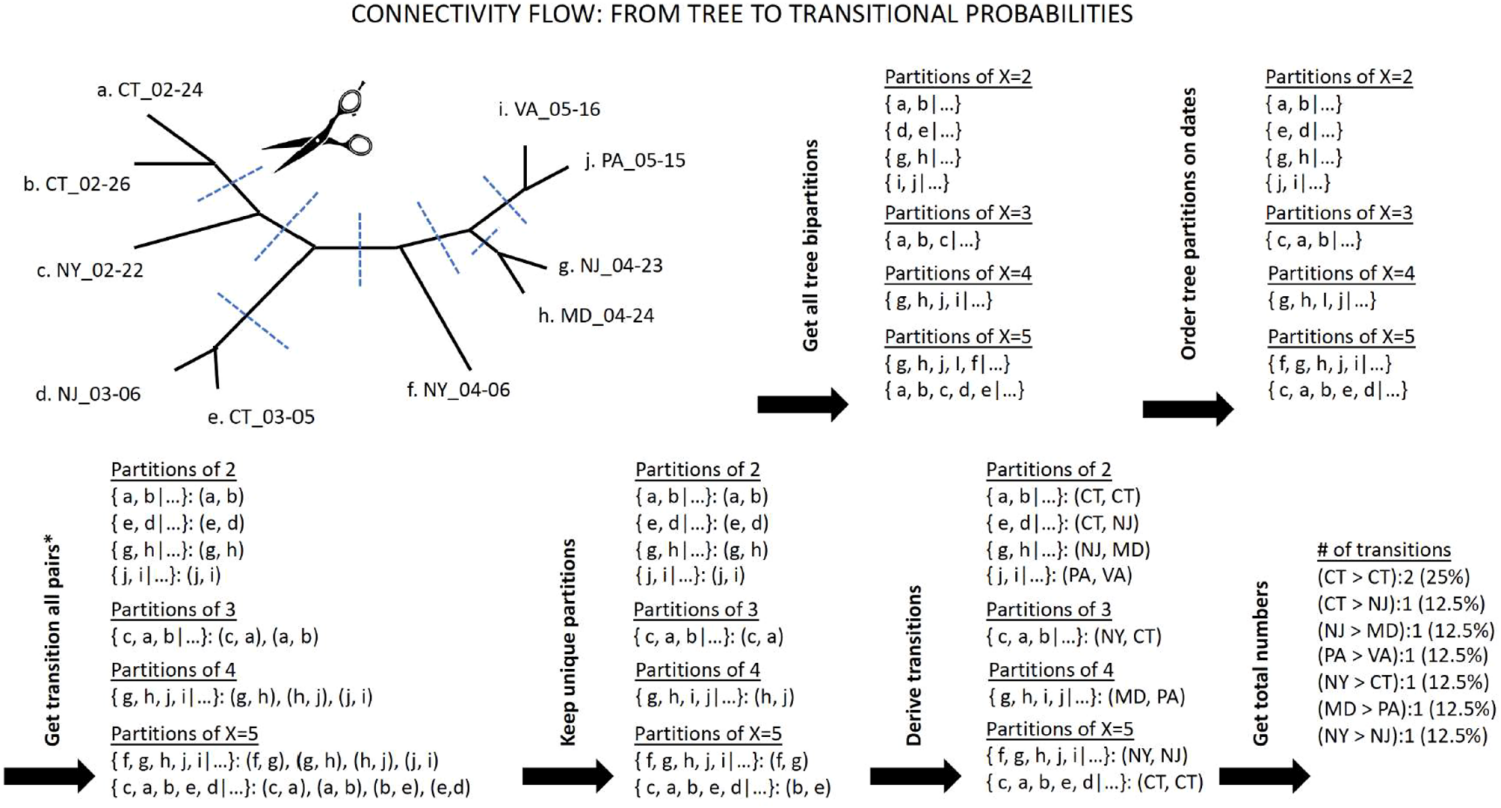
Workflow for estimating genetic connectivity. Here, we use a tree example to explain the workflow that we implemented in order to assign directed connectivity, including incoming, ingrowing, and outgoing connections between each state.

Using custom scripts, we parsed the inferred phylogenetic trees into groups of sequences based on the tree bipartitions. Then, by further parsing the groups in ascending order based on group size (from groups of 2 to X = 10 to preserve rooting information), we determined all unique pairs and state connectivity. To establish directionality between pairs, we used sampling dates for terminal tips and when merging groups of smaller to larger size. For example, we considered the pair (NY-PV09151_USA_NY_2020-03-22, CT-UW-6574_USA_CT_2020-04-03) would be counted as NY → CT, which denotes an incoming transmissional connectivity from NY to CT. Similarly, the pair (NY-PV08434_USA_NY_2020-03-18, NY-NYUMC659_USA_NY_2020-03-18) would be counted as ingrown connectivity NY → NY. Sequences {NY-PV09151_USA_NY_2020-03-22 and CT-UW-6574_USA_CT_2020-04-03} depicted an outgoing connectivity between NY and CT denoted as NY > CT + 1 (Fig. 6). Pair inconsistencies were dropped, and sequences could not be considered as incoming twice. It should be noted that these pairs do not represent direct viral transmissions, but are treated as reflective of an underlying probabilistic aggregated transmission rate. Formally, we define the transmissional genetic connectivity as follows. Having extracted all sequence pairs as above, we built a directed graph $G$ whose nodes $n\in N$ are individual sequences, and whose edges $\left(n,m\right)\in E$ join the extracted sequence pairs, where the time stamp of $n$ precedes that of $m$. Further, we let $f(n)$ denote the geographic state of sequence $n$, and ${N}_{s}={\sum }_{n}f\left(n\right)=s$ denote the total count of sequences belonging to state $s$. Then, we defined the transmissional connectivity rate $T\left({s}_{1},{s}_{2}\right)$ between geographic states ${s}_{1}$ and ${s}_{2}$ as:

$$T\left({s}_{1},{s}_{2}\right)\propto \frac{\left|\left\{\left(n,m\right)\in E|f\left(n\right)={s}_{1},f\left(m\right)={s}_{2}\right\}\right|}{{N}_{{s}_{2}}\cdot {N}_{{s}_{2}}},$$

where the constant of proportionality is calculated such that ${\sum }_{{s}_{2}}T\left({s}_{1},{s}_{2}\right)=1$.

### Estimating geographic distance from initial hotspots

To assign a geographic location for each state, we used the longitude and latitude of the respective largest city. We considered the distance from New York City (NYC, NY), Seattle (Washington) and New Orleans (Louisiana) as the three initial hotspots of the outbreak. The inclusion of New Orleans as a third hotspot did not improve our results, indicating an isolated outbreaking contrast, by removing Louisiana as an outlier, we improved the predictability of the logarithmic curve to R^2^ = 0.4. Calculations were performed using perl scripts (GIS-Distance-0.19, https://github.com/bluefeet/GIS-Distance) freely available under a perl_5 license (Table 1).

**Table 1:** The absolute distance (in km) between major state cities and hotspots.

State	Latitude	Longitude	Distance from NY (Km)	Min [distance from NYC, distance from Seattle, (km)]
New York	40.712776	− 74.005974	0	0
New Jersey	40.735657	− 74.172363	14.24987988	14.24987988
Connecticut	41.179192	− 73.189484	85.97441091	85.97441091
Massachusetts	42.360081	− 71.058884	306.0934983	306.0934983
Louisiana	29.951065	− 90.071533	1880.214437	1880.214437
Michigan	42.332939	− 83.047836	773.5458496	773.5458496
District Of Columbia	38.907192	− 77.036873	327.5658436	327.5658436
Rhode Island	41.82399	− 71.412834	249.4471236	249.4471236
Maryland	39.29044	− 76.612328	272.545463	272.545463
Pennsylvania	39.952583	− 75.165222	129.6091349	129.6091349
Illinois	41.878113	− 87.629799	1144.238847	1144.238847
Indiana	39.768402	− 86.158066	1035.902562	1035.902562
Colorado	39.739235	− 104.99025	2618.92694	1640.656701
Washington	47.606209	− 122.332069	3865.343137	0
Delaware	39.739071	− 75.539787	169.3360522	169.3360522
Georgia	33.748997	− 84.387985	1200.258419	1200.258419
Mississippi	42.247452	− 84.408852	882.6192762	882.6192762
Vermont	44.47599	− 73.211	423.453307	423.453307
Nevada	36.169941	− 115.139832	3584.696039	1402.54933
Ohio	41.499321	− 81.694359	649.8243383	649.8243383
Florida	27.950575	− 82.457176	1615.374262	1615.374262
Virginia	36.84513	− 75.97544	462.6503612	462.6503612
Oklahoma	35.46756	− 97.516426	2131.172228	2131.172228
Wisconsin	43.038902	− 87.906471	1178.047729	1178.047729
Kentucky	38.252666	− 85.758453	1044.090281	1044.090281
Missouri	38.7269	− 94.71842	1780.925619	1780.925619
Alabama	33.522861	− 86.807701	1385.944612	1385.944612
California	37.774929	− 122.419418	4128.890575	1093.158971
Kansas	37.68602	− 97.335571	2031.992059	2031.992059
New Hampshire	42.990929	− 71.463089	329.388085	329.388085
New Mexico	35.0844444	− 106.6505556	2914.047894	1903.828858
Arizona	32.25346	− 110.911789	3403.255018	1960.423336
Minnesota	44.977753	− 93.265015	1635.581625	1635.581625
Maine	43.659222	− 70.256523	450.1866372	450.1866372
Idaho	43.615021	− 116.202316	3455.117344	650.9714118
Iowa	41.586834	− 93.624962	1641.926903	1641.926903
South Carolina	32.77647	− 79.93103	1027.66482	1027.66482
North Carolina	35.223789	− 80.841141	854.7086843	854.7086843
Tennessee	36.162663	− 86.781601	1220.705852	1220.705852
Nebraska	41.256538	− 95.934502	1836.683114	1836.683114
Texas	29.760427	− 95.369804	2281.234344	2281.234344
Oregon	45.51223	− 122.658722	3924.005523	234.1635691
North Dakota	46.876961	− 96.784637	1943.898966	1921.486278
West Virginia	38.351189	− 81.638359	704.9255916	704.9255916
Arkansas	34.746483	− 92.289597	1735.09253	1735.09253
Montana	45.783287	− 108.500687	2827.009373	1072.597358
Wyoming	41.14024	− 104.818802	2575.395552	1561.722056
Alaska	61.218056	− 149.900284	5409.611693	2308.628727
Utah	40.75848	− 111.888138	3166.306285	1126.553692
South Dakota	43.54731	− 96.7313	1894.123012	1894.123012
Hawaii	19.5555	− 154.879852	7853.670071	4279.36227

Based on longitude and latitude (columns 2 and 3) we calculated the distance between each states’ largest city (in population) and New York City (column 4) and the minimum distance between each states’ largest city and either New York City (NYC) or Seattle (WA) (column 5).

### Maximum reproduction rate R_t_

To calculate the maximum reproduction rate R_t_, we used the maximum R_t_ value for each state from ‘https://rt.live/us/’^42^ (https://github.com/rtcovidlive/covid-model) during the first wave of the pandemic (through August 2020). R_t_ represents the effective reproduction rate of the virus calculated for each locale but different studies have developed alternative methods for calculating maximum reproduction and transmission rates based on demographic and epidemiological data^43–46^. This value allows us to estimate how many secondary infections are likely to occur from a single infection in a specific area (Table 2).

**Table 2:** Maximum Reproduction Rates (maximum R_t_) per state and average maximum R_t_ from neighboring states per state.

States	Maximum reproduction rate R_t_	Neighboring maximum R_t_ (average)
New York	5.3	3.3
New Jersey	4	3.32
Connecticut	3.1	3.53
Massachusetts	2.8	2.88
Maryland	2.9	2.66
Pennsylvania	3.2	3.25
Virginia	2.4	2.45
Delaware	2	
North Carolina	2.5	
West Virginia	1.7	
Tennessee	2.7	

In this table, we show the maximum R_t_ per state and the average maximum R_t_ from all neighboring states (per state) as provided from *rt.live* covid-model from the beginning of the first wave through August 2020*.*

### Urbanization index

As an indication of how “urban” a state is, we used the urbanization index and data as defined and provided by FiveThirtyEight (‘https://github.com/fivethirtyeight/data/tree/master/urbanization-index’). FiveThirtyEight’s urbanization index is calculated as the natural logarithm of the average number of people living within a five-mile radius of a given resident (Table 3).

**Table 3:** Urbanization Index.

State	Urbanization index
New York	12.56
New Jersey	12.24
Connecticut	11.41
Massachusetts	11.84
Maryland	11.71
Pennsylvania	11.15
Virginia	10.91

How “Urban” is each state, as calculated by FiveThirtyEight and included in our analysis as Urbanization Index (U).

### Regression analysis models

We performed multiple linear regression analyses in order to assess the importance of each factor on the prediction of the per-state death rate. We used epidemiological data from seven states (NY, CT, MA, PA, NJ, VA, and MD), over a series of ten time points from April 29 to July 23, 2020. We regressed the per-state death rate (the cumulative ratio of deaths per million from the earliest date) on either three variables (transmission rate (R0), urbanization index, distance from NYC) or six variables (transmission rate (R0), urbanization index, distance from NYC, ingoing/outgoing/ingrowing rates per state). Prior to the analysis, we calculated Z-scores for all variables (enforcing zero mean and unit covariance). For distance from NYC, we used either the geographic distance between the state’s capital and NYC, or the transmissional distance as defined below. For each model, we calculated the log-likelihood by fitting a variance parameter to the predicted outputs and using a Gaussian noise model. Hence, we set ${{\sigma }_{t}}^{2}=(1/N){\Sigma }_{i=1:N}({y}_{it}-{\beta }_{{\varvec{t}}}{{\varvec{x}}}_{{\varvec{i}}{\varvec{t}}}{)}^{2}$, where $N$ is the number of states, ${\beta }_{t}$ and ${x}_{it}$ are the vectors of coefficients and features associated with state *i* at time *t* respectively, and ${y}_{it}$ is the associated death rate. We calculated the log-likelihood at time *t* as ${L}_{t}={\Sigma }_{i}log(Gauss({y}_{it}-{\beta }_{{\varvec{t}}}{{\varvec{x}}}_{{\varvec{i}}{\varvec{t}}};0,{{\varvec{\sigma}}}_{{\varvec{t}}\boldsymbol{ }}))$, where *Gauss* is the probability density function of a normal distribution. We then compared the log-likelihood differences of pairs of models over time using Pearson’s correlation coefficient (differences versus temporal ordering).

### Random walk model

We define the transmissional-distance of a state from NYC as the expected first arrival time at that state of a Markov random walk starting at NYC, using the transmissional probabilities between states inferred from the phylogenetic analysis. Hence, we set ${d}_{ij}=E(min(\{t|{s}_{t}=j\})|{s}_{0}=i)$ for the directed transmission-distance between states i and j (which is not a metric), where ${s}_{t}=i$ indicates that the state at time *t* in a sampled random walk is *i,* and *E*(.) denotes expectation. To estimate these distances, we ran 1000 such random walks for 1000 time-steps and used the empirical mean time of first arrival at each state across samples. As above, we calculated Z-scores for the resulting distances for each state.

### Mitigation analysis

In order to model severely restricted mobility to represent implemented interventions between geographically adjacent states ${s}_{1}$ and ${s}_{2}$, we set a reduction factor $r=0.1$, and updated the transmission probabilities as: ${P}^{^{\prime}}\left({s}_{b}|{s}_{a}\right)=r\cdot P\left({s}_{b}|{s}_{a}\right)$, and ${P}^{^{\prime}}\left({s}_{a}|{s}_{a}\right)=P\left({s}_{a}|{s}_{a}\right)+(1-r)\cdot P\left({s}_{b}|{s}_{a}\right)$, where ${P}^{^{\prime}}\left({s}_{a}|{s}_{b}\right)$ is the updated transmissional probability between states ${s}_{a}$ and ${s}_{b}$. We made such updates for $a=1, b=2,$ and $a=2, b=1$ simultaneously, hence restricting the connectivity in both directions. We then recalculated the distances ${d}_{ij}^{{s}_{1}{s}_{2}}$, i.e., the distance between states i and j, given the link between ${s}_{1}$ and ${s}_{2}$ has been restricted. We then used these to estimate the overall predicted reduction in death rate given the intervention as: ${\Delta }_{{s}_{1}{s}_{2}}={\sum }_{it}{w}_{i}\cdot ({y}_{it}^{^{\prime}}-{y}_{it})$, where ${w}_{i}$ is a weighting factor proportional to the population of state $i$ (and ${\sum }_{i}{w}_{i}=1$), and ${y}_{it}^{^{\prime}}$ is the predicted death rate for state i at time t when ${d}_{ij}^{{s}_{1}{s}_{2}}$ is substituted for ${d}_{ij}$ in the predictive model from the regression analysis. For the general lockdown comparison, we apply the reduction factor, $r$, as above, to the connections between all pairs of geographically adjacent states and renormalize the transmission probability matrix by increasing the ingrowing probability for each state. The ‘degree of lockdown’ is defined as $f = 1- r$. We recalculated the distances between states and estimate the overall predicted reduction in death rate using the same method as above. For comparison, we find the matching degree of full lockdown degree closest to the change in death rate for each of the single link restrictions, as well as the most effective two-link restriction, which occurs when NY-PA and MD-PA links are cut (using $r = 0.1$ for single and paired cut links).

## Supplementary Information

Supplementary Legends.

Supplementary Figures.

## Data Availability

The datasets used in this study are available in public databases. SARS-CoV-2 genomes were retrieved from the GISAID (https://gisaid.org/) database^2^. Accession IDs, originating laboratories, and submitting laboratories for each state are provided in Data s1–7. Epidemiological data concerning the daily and total deaths per million individuals were retrieved from Worldometer ‘worldometers.info/coronavirus/’. Maximum reproduction rates were retrieved from The COVID Tracking Project at “https://covidtracking.com/” and ‘https://rt.live/us/’^42^, but different studies have developed alternative methods for calculating maximum reproduction and transmission rates based on demographic and epidemiological data^43–46^.
