# Supplementary material for: Genetic determination of regional connectivity in modelling the spread of COVID-19 outbreak for more efficient mitigation strategies
Source: Sci Rep. 2023 May 25;13:8470. doi: 10.1038/s41598-023-34959-2 (PMC10209930; doi:10.1038/s41598-023-34959-2)
Supplement: Supplementary file 1 — Supplementary Legends. [file 41598_2023_34959_MOESM1_ESM.docx]

**Supplementary figure S1. New York outbreak.** Using BEAST v.2.6.3 (https://www.beast2.org/) we performed a Bayesian phylogenetic analysis for all sequences from New York under a Yule process. The phylogenetic tree was visualized using FigTree v1.4.4 (http://tree.bio.ed.ac.uk/software/figtree/). New York sequences were colored yellow, while world reference sequences were colored black.

**Supplementary figure S2. New Jersey outbreak.** Using BEAST v.2.6.3 (https://www.beast2.org/) we performed a Bayesian phylogenetic analysis for all sequences from New Jersey under a Yule process. The phylogenetic tree was visualized using FigTree v1.4.4 (http://tree.bio.ed.ac.uk/software/figtree/). New Jersey sequences were colored red, while world reference sequences were colored black.

**Supplementary figure S3. Connecticut outbreak.** Using BEAST v.2.6.3 (https://www.beast2.org/) we performed a Bayesian phylogenetic analysis for all sequences from Connecticut under a Yule process. The phylogenetic tree was visualized using FigTree v1.4.4 (http://tree.bio.ed.ac.uk/software/figtree/). Connecticut sequences were colored blue, while world reference sequences were colored black.

**Supplementary figure S4. Massachusetts outbreak.** Using BEAST v.2.6.3 (https://www.beast2.org/) we performed a Bayesian phylogenetic analysis for all sequences from Massachusetts under a Yule process. The phylogenetic tree was visualized using FigTree v1.4.4 (http://tree.bio.ed.ac.uk/software/figtree/). Massachusetts sequences were colored cyan, while world reference sequences were colored black.

**Supplementary figure S5. Pennsylvania outbreak.** Using BEAST v.2.6.3 (https://www.beast2.org/) we performed a Bayesian phylogenetic analysis for all sequences from Pennsylvania under a Yule process. The phylogenetic tree was visualized using FigTree v1.4.4 (http://tree.bio.ed.ac.uk/software/figtree/). Pennsylvania sequences were colored orange, while world reference sequences were colored black.

**Supplementary figure S6. Maryland outbreak.** Using BEAST v.2.6.3 (https://www.beast2.org/) we performed a Bayesian phylogenetic analysis for all sequences from Maryland under a Yule process. The phylogenetic tree was visualized using FigTree v1.4.4 (http://tree.bio.ed.ac.uk/software/figtree/). Maryland sequences were colored green, while world reference sequences were colored black.

**Supplementary figure S7. Virginia outbreak.** Using BEAST v.2.6.3 (https://www.beast2.org/) we performed a Bayesian phylogenetic analysis for all sequences from Virginia under a Yule process. The phylogenetic tree was visualized using FigTree v1.4.4 (http://tree.bio.ed.ac.uk/software/figtree/). Virginia sequences were colored brown, while world reference sequences were colored black.

**Supplementary figure S8. Model feature coefficients and likelihood.** In i) we see the importance of our model’s feature coefficients. U and D showed high significance throughout the entire first wave, while the use of Rt showed greater significance at the beginning of the outbreak but eventually decreased. In ii) by including transmissional-distance (Dt), we were able to significantly increase our model’s predictive power throughout the first wave compared to our baseline model (p=0.0003). The figures and models were created using *R* *v4.1.2.*

**Supplementary figure S9. Selected world references spanning the Nextstrain first-wave tree.** We depict the selected world reference sequences in a higher resolution tree. Nextstrain tree was inferred using *nextstrain/ncov (*[*https://github.com/nextstrain/ncov*](https://github.com/nextstrain/ncov)*)*. 50 sequences were manually selected spanning all Nextstrain lineages 19A, 19B, 20A, 20B, and 20C. The majority (76%) of the selected world reference sequences represent early infections, occurring between December 2019 - April 2020 in order to consider the pandemic’s early divergence profile.

**Supplementary figure S10. Selected New York (NY) sequences spanning the NY outbreak.** Using BEAST v.2.6.3 (https://www.beast2.org/) we performed a Bayesian phylogenetic analysis for all sequences from NY. The phylogenetic tree was visualized using FigTree v1.4.4 (http://tree.bio.ed.ac.uk/software/figtree/). Yellow stars were manually added using Adobe Illustrator 2022 (<https://www.adobe.com/products/illustrator.html>) and denote the nearest node position for every sequence (in yellow) used to estimate genetic connectivity.

**Supplementary figure S11. Selected Connecticut (CT) sequences spanning the CT outbreak.** Using BEAST v.2.6.3 (https://www.beast2.org/) we performed a Bayesian phylogenetic analysis for all sequences from CT. The phylogenetic tree was visualized using FigTree v1.4.4 (http://tree.bio.ed.ac.uk/software/figtree/). Yellow stars were manually added using Adobe Illustrator 2022 (<https://www.adobe.com/products/illustrator.html>) and denote the nearest node position for every sequence (in blue) used to estimate genetic connectivity.

**Supplementary figure S12. Selected Massachusetts (MA) sequences spanning the MA outbreak.** Using BEAST v.2.6.3 (https://www.beast2.org/) we performed a Bayesian phylogenetic analysis for all sequences from MA. The phylogenetic tree was visualized using FigTree v1.4.4 (http://tree.bio.ed.ac.uk/software/figtree/). Yellow stars were manually added using Adobe Illustrator 2022 (<https://www.adobe.com/products/illustrator.html>) and denote the nearest node position for every sequence (in cyan) used to estimate genetic connectivity.

**Supplementary figure S13. Selected Virginia (VA) sequences spanning the VA outbreak.** Using BEAST v.2.6.3 (https://www.beast2.org/) we performed a Bayesian phylogenetic analysis for all sequences from VA. The phylogenetic tree was visualized using FigTree v1.4.4 (http://tree.bio.ed.ac.uk/software/figtree/). Yellow stars were manually curated using Adobe Illustrator 2022 (<https://www.adobe.com/products/illustrator.html>) and denote the nearest node position for every sequence (in brown) used to estimate genetic connectivity.

**Supplementary figure S14. Evolutionary divergence comparison for total versus selected sequences comparison.** We calculated the number of base differences per sequence from averaging over all sequence pairs. Standard error estimates were obtained by a bootstrap procedure of 100 replicates. Evolutionary analyses were conducted in MEGA11 (<https://www.megasoftware.net/>). Bar plot was created using Microsoft Excel v16.46 (<https://www.microsoft.com/en-us/microsoft-365/excel>).
