# Supplementary material for: Genetic determination of regional connectivity in modelling the spread of COVID-19 outbreak for more efficient mitigation strategies
Source: Sci Rep. 2023 May 25;13:8470. doi: 10.1038/s41598-023-34959-2 (PMC10209930; doi:10.1038/s41598-023-34959-2)

figure S1.NY outbreak

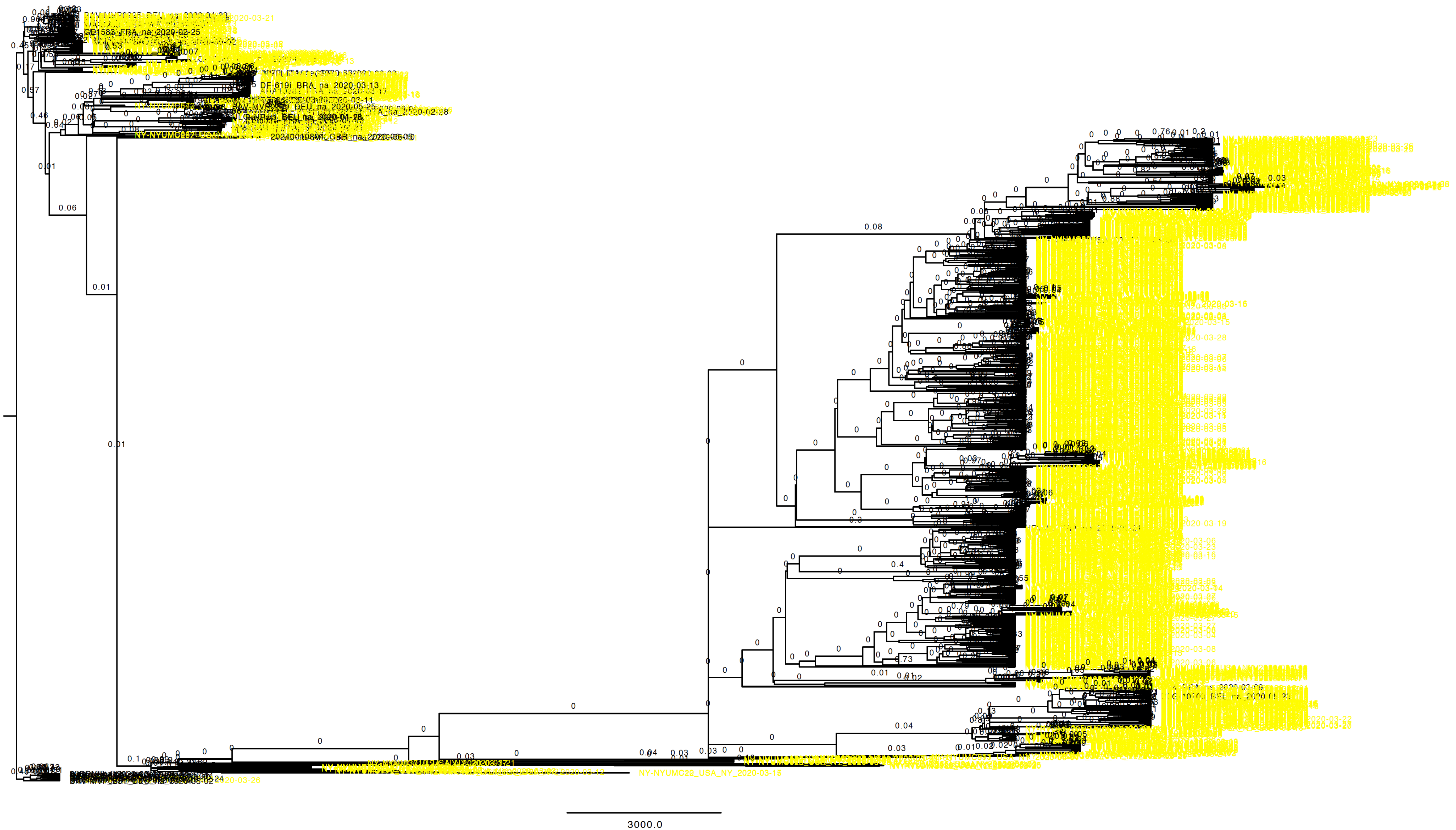

figure S2.NJ outbreak

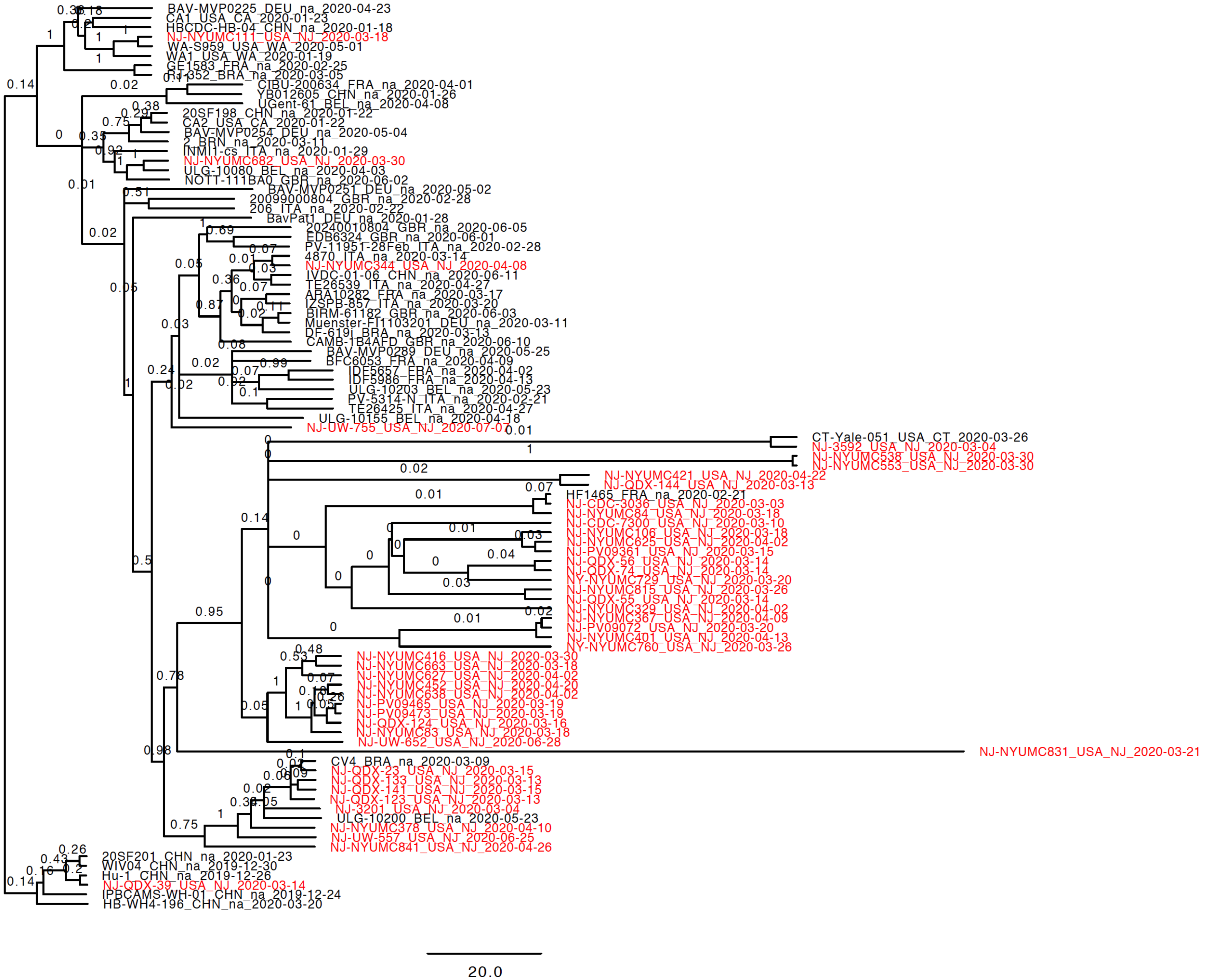

figure S3. CT outbreak

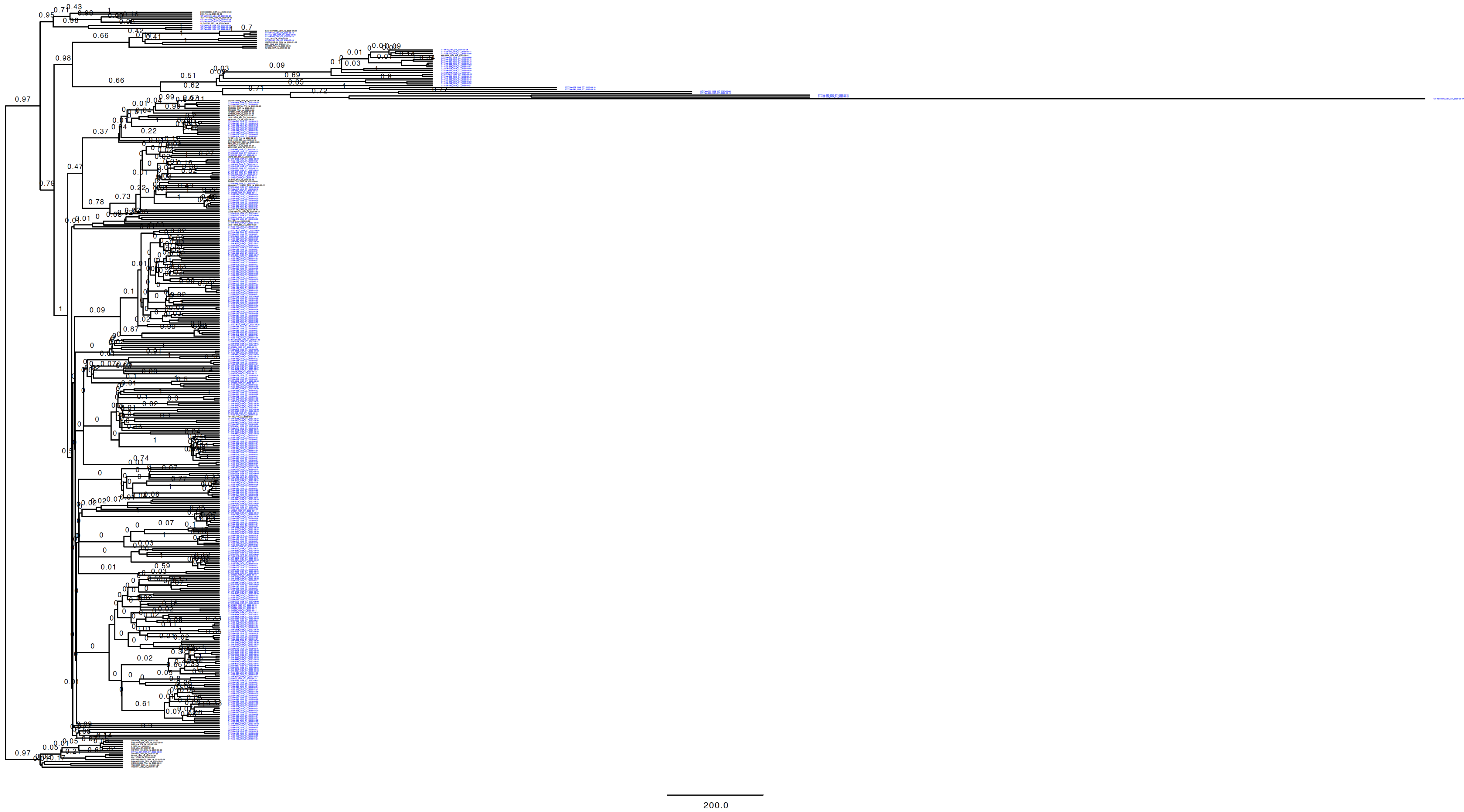

figure S4. MA outbreak

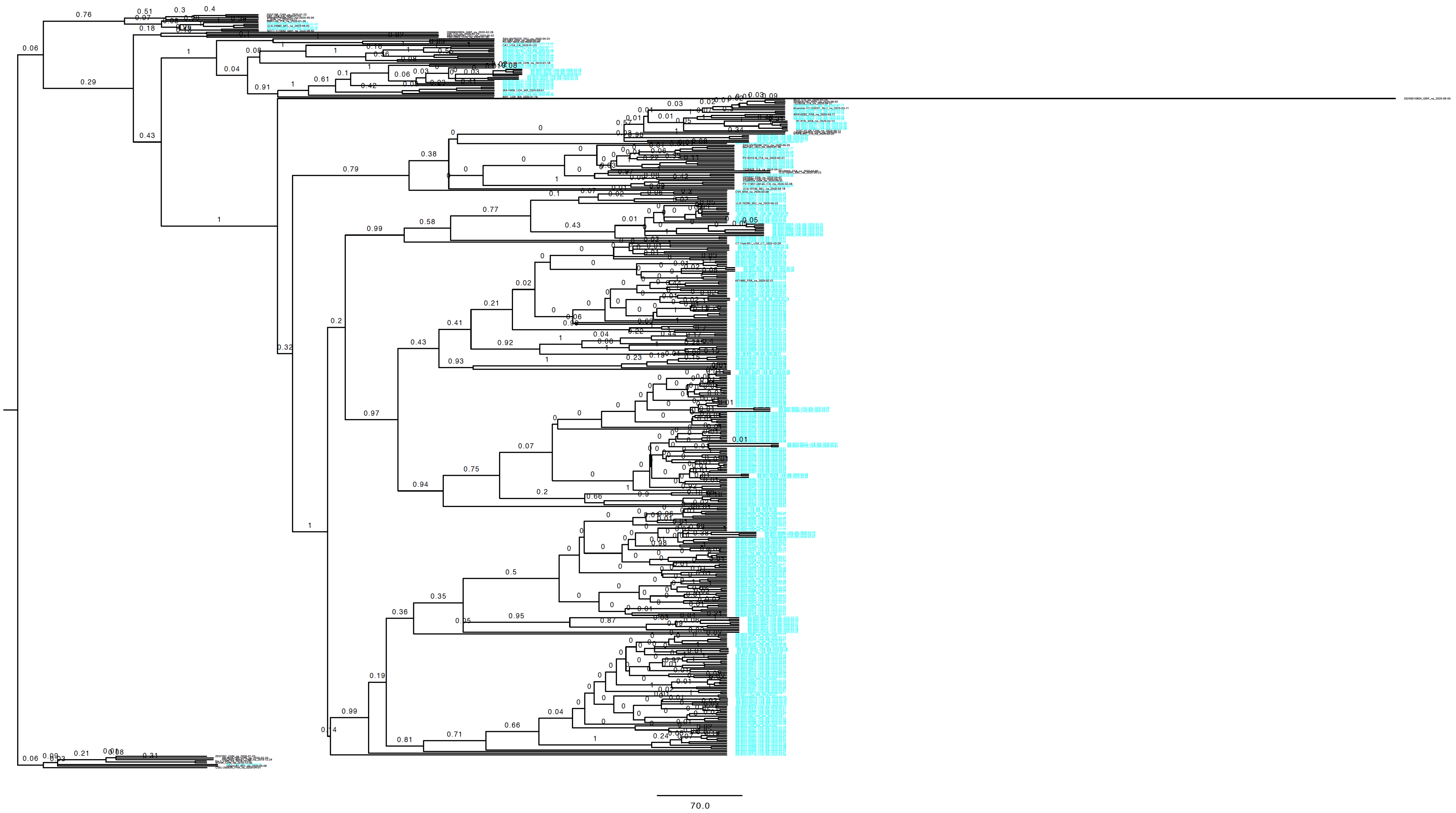

figure S5. PA outbreak

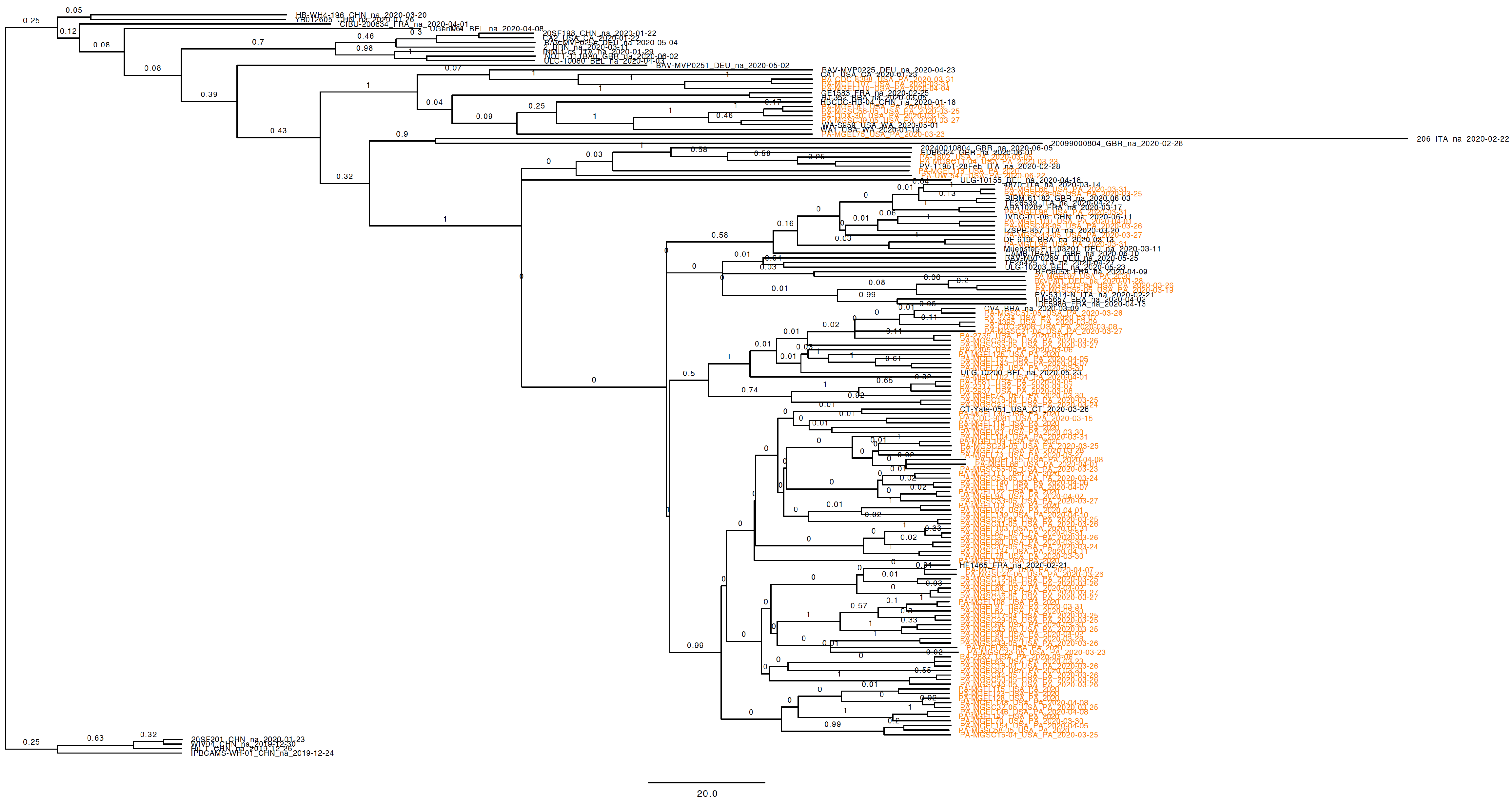

figure S6. MD outbreak

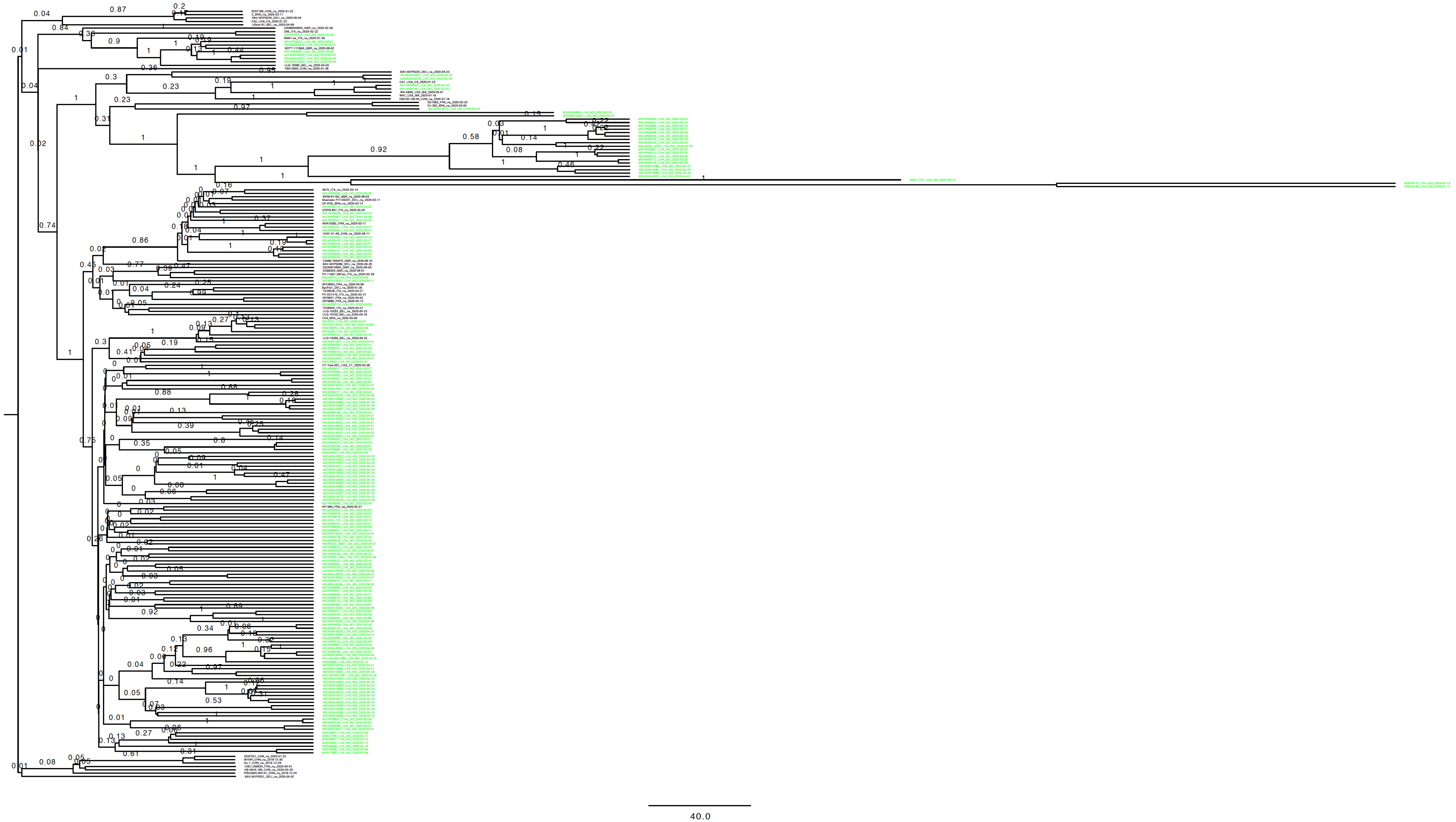

figure S7. VA outbreak

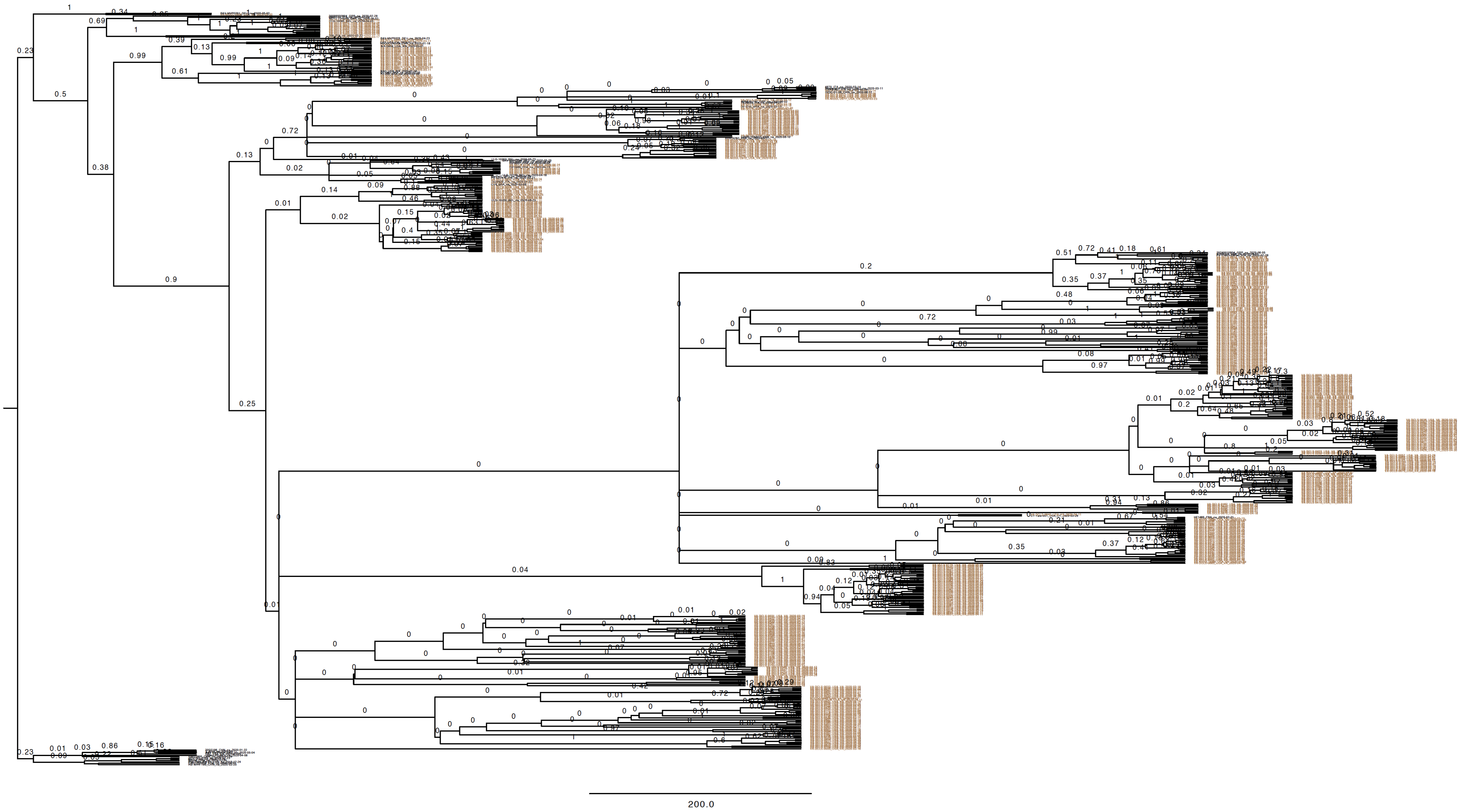

figure S8. Model Likelihood

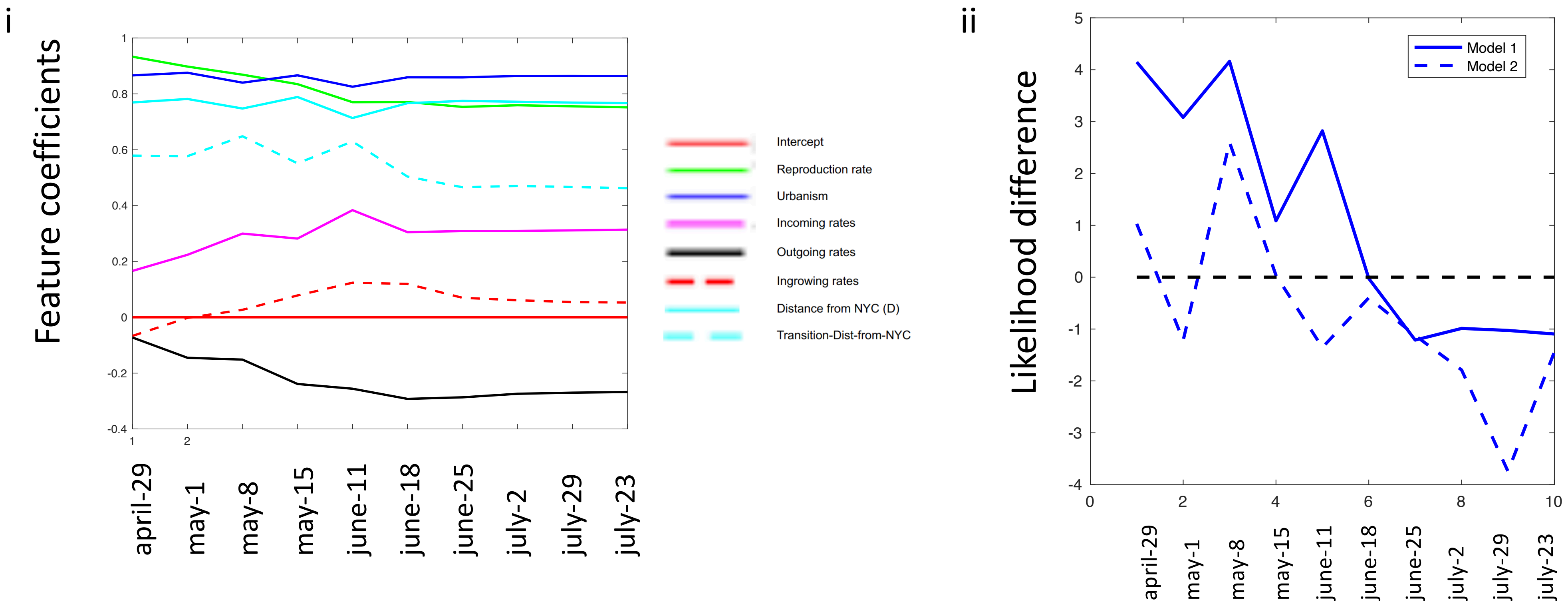

figure S9. World reference sequences

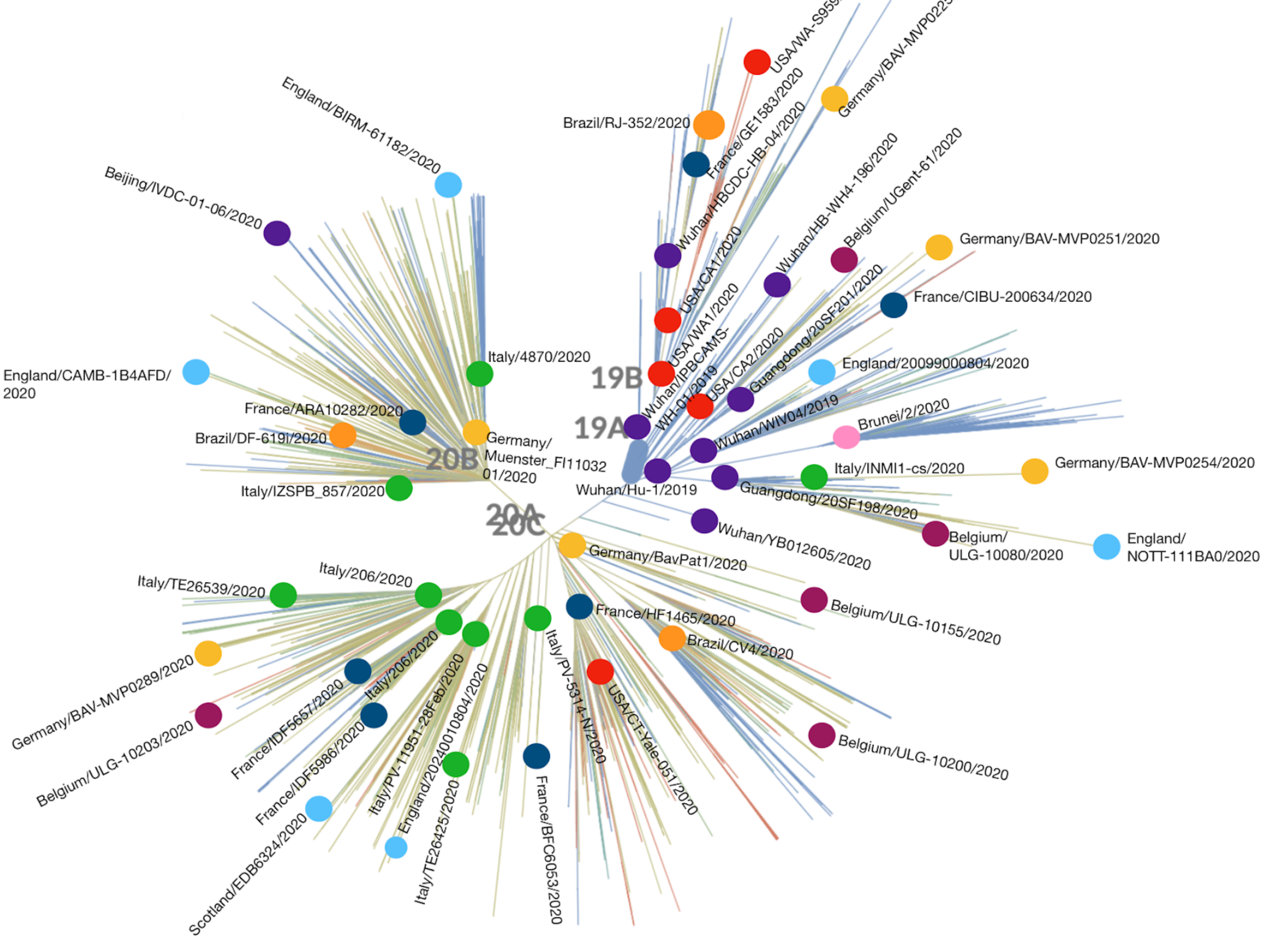

figure S10.NY outbreak – selected state sequences

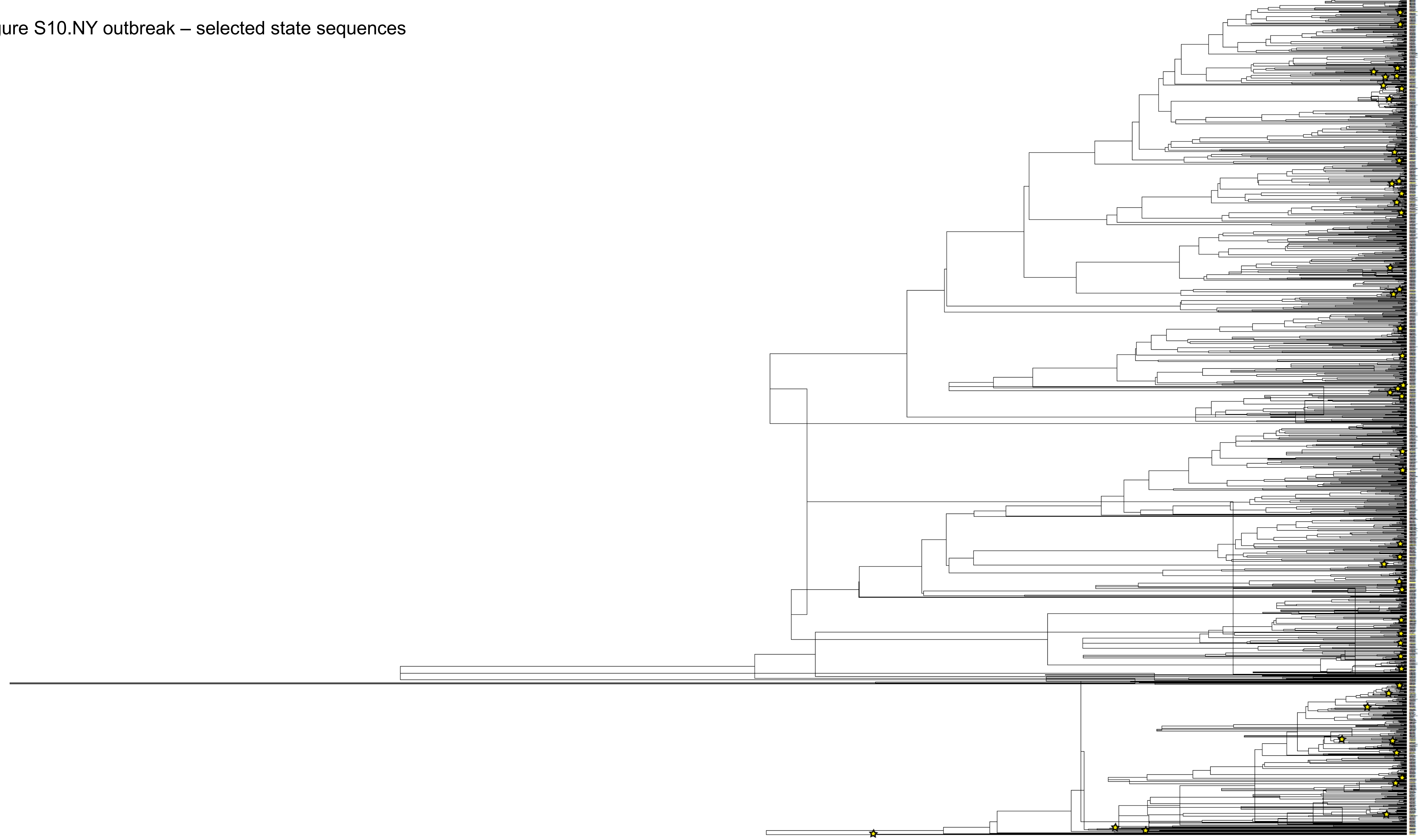

figure S11.CT outbreak – selected state sequences

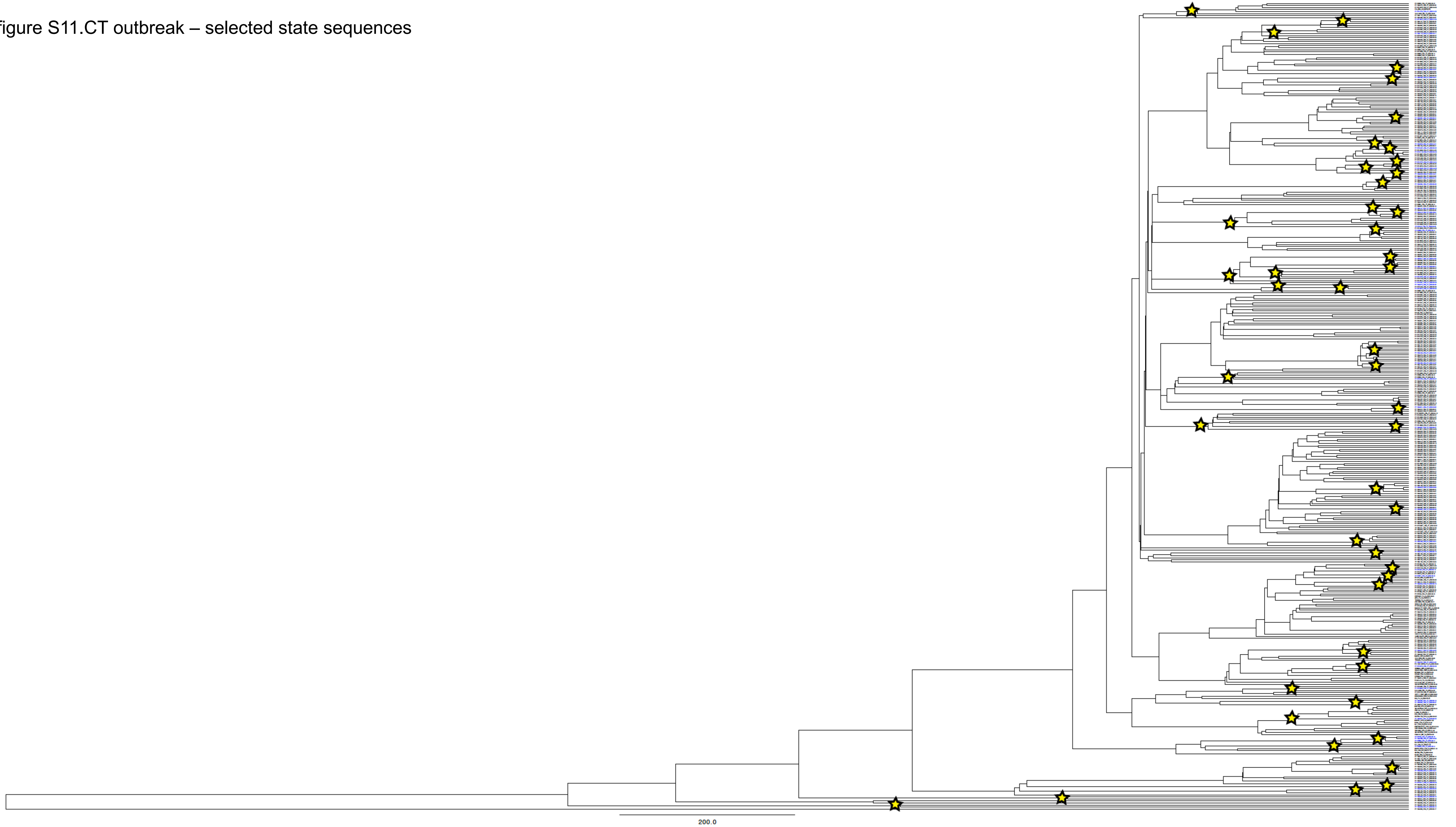

figure S12.MA outbreak – selected state sequences

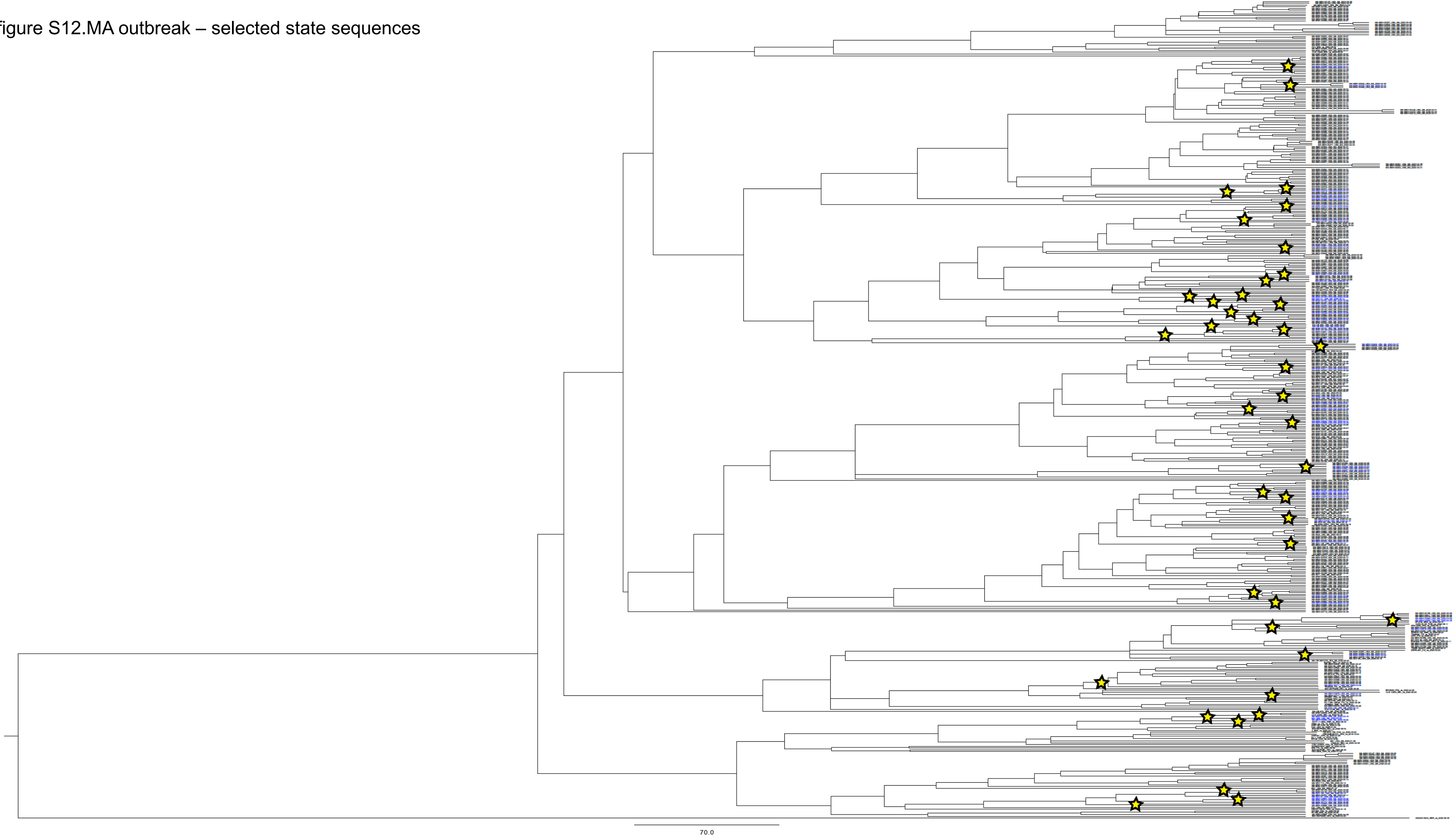

figure S13.VA outbreak – selected state sequences

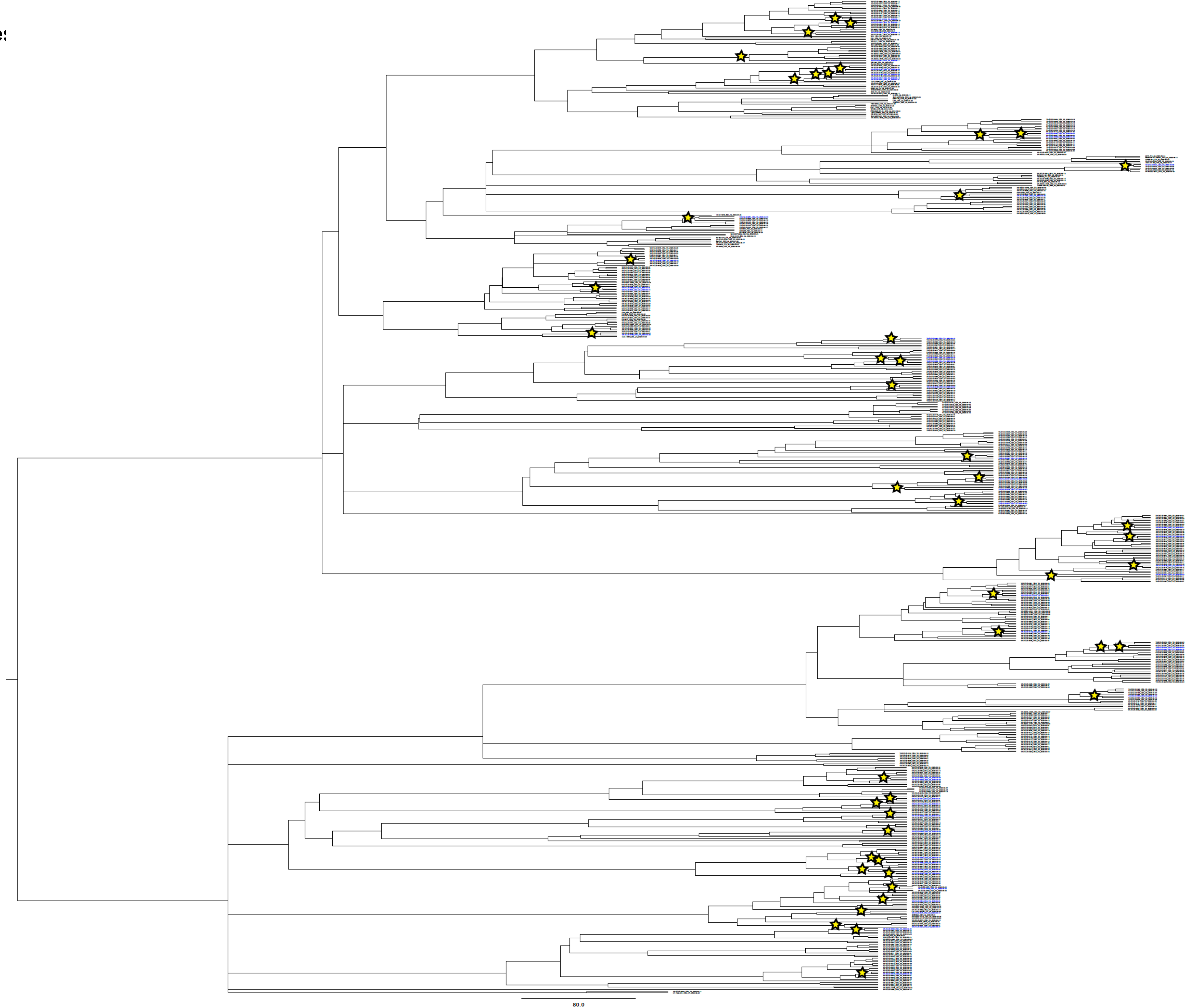

figure S14. Estimates of average evolutionary divergence

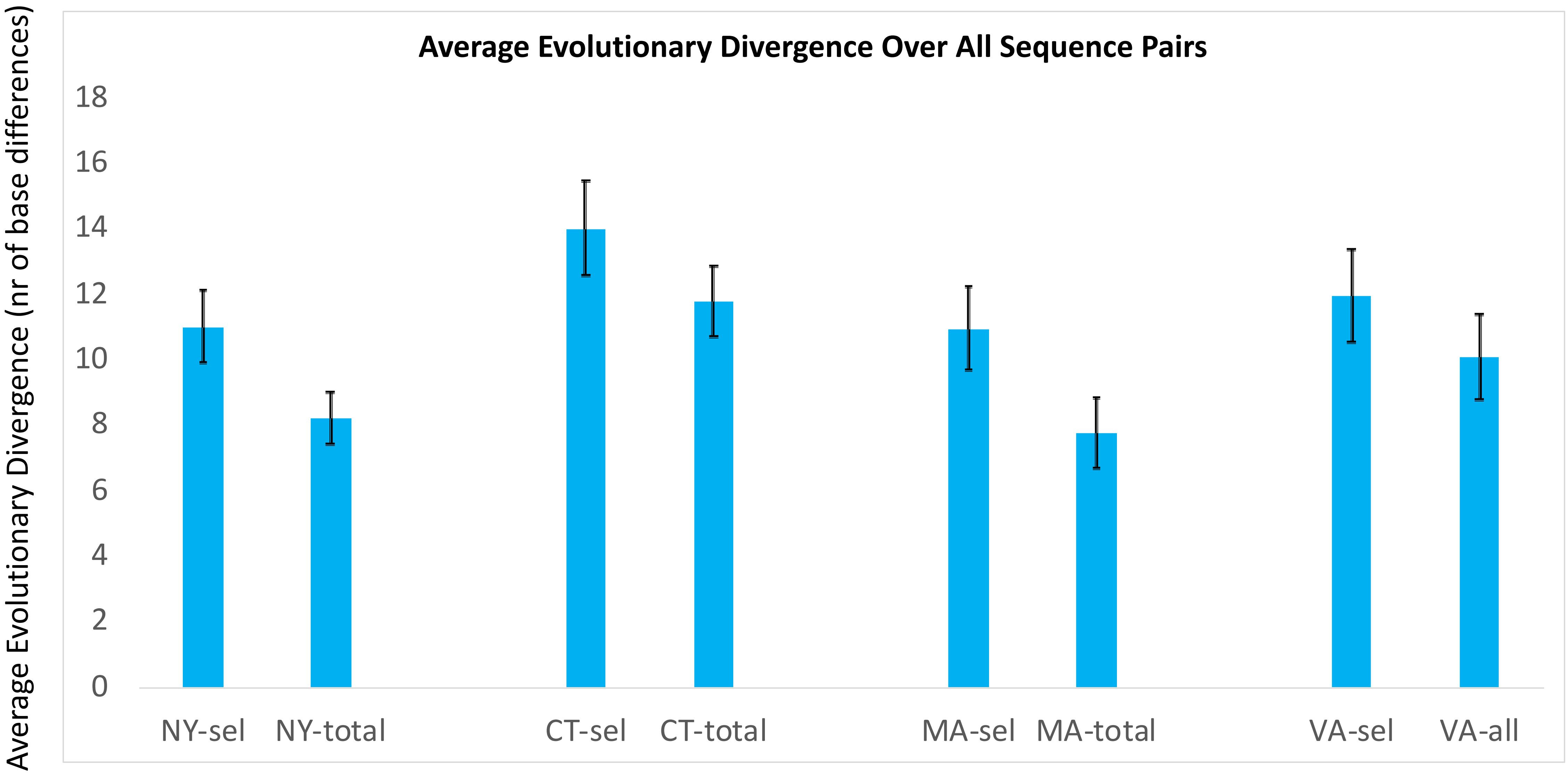

Supplement: Supplementary file 2 — Supplementary Figures. [file 41598_2023_34959_MOESM2_ESM.pdf]
